# An efficient and facile access to highly functionalized pyrrole derivatives

**DOI:** 10.3762/bjoc.14.75

**Published:** 2018-04-20

**Authors:** Meng Gao, Wenting Zhao, Hongyi Zhao, Ziyun Lin, Dongfeng Zhang, Haihong Huang

**Affiliations:** 1 State Key Laboratory of Bioactive Substance and Function of Natural Medicines & Beijing Key Laboratory of Active Substance Discovery and Druggability Evaluation, Institute of Materia Medica, Peking Union Medical College and Chinese Academy of Medical Sciences, 1 Xian Nong Tan Street, Beijing 100050, China

**Keywords:** azomethine ylides, 1,3-dipolar cycloaddition, one-pot synthesis, polysubstituted pyrroles, pyrrolo[3,4-*c*]pyrrole-1,3-diones

## Abstract

A straightforward and one-pot synthesis of pyrrolo[3,4-*c*]pyrrole-1,3-diones via Ag(I)-catalyzed 1,3-dipolar cycloaddition of azomethine ylides with *N*-alkyl maleimide, followed by readily complete oxidation with DDQ, has been successfully developed. Further transformation with alkylamine/sodium alkoxide alcohol solution conveniently afforded novel polysubstituted pyrroles in good to excellent yields. This methodology for highly functionalized pyrroles performed well over a broad scope of substrates. It is conceivable that this efficient construction method for privileged pyrrole scaffolds could deliver more active compounds for medicinal chemistry research.

## Introduction

**Figure 1:**
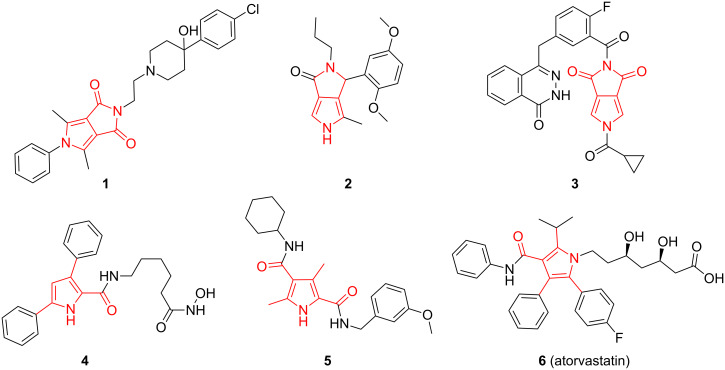
Representative pyrrolo[3,4-*c*]pyrrole-1,3-diones and polysubstituted pyrrole derivatives.

Pyrroles are an important class of five-membered nitrogen containing heterocycles which are widely used in numerous medically relevant fields. Pyrrolo[3,4-*c*]pyrrole-1,3-diones and highly substituted pyrroles with amide groups are important frameworks of pyrrole compounds that play important roles in medicinal chemistry (Figure 1), such as analgesic agent **1** [1], BET bromodomain inhibitor **2** [2], selective PARP-1 inhibitor **3** [3], histone deacetylase inhibitor with antitumor activity **4** [4], *Flavivirus* inhibitor **5** [5], and for treating cardiovascular diseases (atorvastatin, **6**) [6].

A number of methods have been reported till now to synthesize pyrroles and pyrrole containing analogs [6]. In the past decade, 1,3-dipolar cycloaddition has become a fundamental synthetic method for the construction of nitrogen-containing five-membered heterocycles including pyrroles. It is worth to note that considerable efforts have been expended to build stereogenic centers of pyrrolidine derivatives using chiral catalysts [7].

**Scheme 1:**
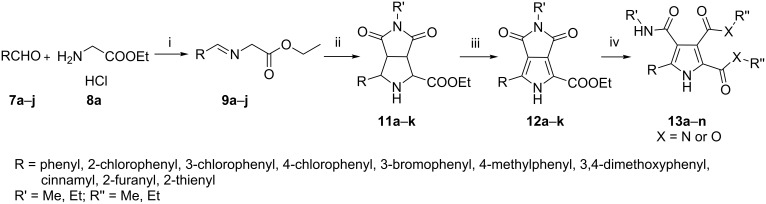
Synthetic pathway for the preparation of pyrrolo[3,4-*c*]pyrrole-1,3-diones and highly substituted pyrroles. Reagents and conditions: (i) Et_3_N, MgSO_4_, CH_2_Cl_2_, rt, 8 h; (ii) AgOAc, *N*-methylmaleimide (**10a**) or *N*-ethylmaleimide (**10b**), CH_2_Cl_2_, rt, 3 h; (iii) DDQ, toluene, rt, 12–80 h; (iv) a: MeNH_2_ or EtNH_2_ in EtOH, 80 °C, in a sealed tube for 3 h; b: MeONa/MeOH, rt, 24 h or EtONa/EtOH, rt, 36 h.

As our aim was to construct a small library of polysubstituted pyrroles for antibacterial screening, it prompted us to develop a concise and efficient synthesis of pyrrolo[3,4-*c*]pyrrole-1,3-diones [8–10] via 1,3-dipolar cycloaddition without any chiral catalysts/ligands and a facile access to highly substituted pyrroles with amide groups or ester groups. Herein, we propose a straightforward and one-pot synthesis of pyrrolo[3,4-*c*]pyrrole-1,3-diones via Ag(I)-catalyzed 1,3-dipolar cycloaddition of azomethine ylides with *N*-alkyl maleimide, followed by a facile oxidation using DDQ as oxidant. Further manipulation with alkylamine/sodium alkoxide alcohol solution conveniently led to novel polysubstituted pyrroles in good to excellent yields (Scheme 1).

## Results and Discussion

As shown in Scheme 1, one advantage of this 1,3-dipolar cycloaddition for constructing privileged pyrrole scaffolds is the broad scope of azomethine ylides as substrates. To our surprise, although there were many reports [11–16] about the synthesis of ethyl *N*-(phenylmethylidene)glycinate (**9a**, azomethine ylide) as the key substrate for 1,3-dipolar cycloadditions, the reaction conditions were very different exemplified by the wide range of reaction times from 0.3 h to 120 h, and the variable yields from 68% to 100% in different solvents. In order to establish the optimal experimental conditions suitable for the one-pot synthesis of pyrrolo[3,4-*c*]pyrrole-1,3-diones **12a**–**k**, we investigated the reaction conditions step by step.

Initially, benzaldehyde (**7a**) and ethyl glycinate hydrochloride (**8a**) were chosen as the model substrates for obtaining azomethine ylide **9a**, and the results are summarized in Table 1. Based on reported methods, Et_3_N as base, MgSO_4_ as desiccant and CH_2_Cl_2_ as solvent were used for the synthesis of the azomethine ylide [13–14,17–24]. Because **9a** easily decomposed to benzaldehyde on silica gel when monitored by TLC, we used ^1^H NMR to monitor the reaction process. Table 1 shows that the reaction time was crucial to the extent of the reaction. **9a** could be obtained in excellent yield (94% total yield of **7a** and **9a**, of which 98% was **9a**) without any further purification when the reaction time was prolonged to 8 h. The outcome of a 8 h reaction is favorable compared to the results of 2 h and 4 h reaction time (Table 1, entry 3 vs entries 1 and 2).

**Table 1:** Optimization of the reaction conditions for the synthesis of the azomethine ylide^a^.

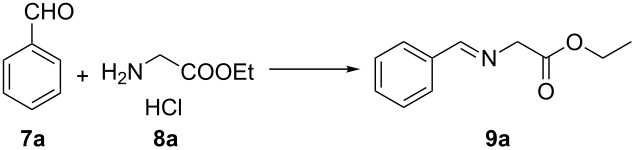			

entry	time	yield (%)^b^	ratio of **9a**/**7a**^c^

1	2 h	99	12:1
2	4 h	98	24:1
3	8 h	94	47:1

^a^General reaction conditions: benzaldehyde (2.5 mmol), ethyl glycinate hydrochloride (3.75 mmol), Et_3_N (3.75 mmol), and MgSO_4_ (3.75 mmol), rt. ^b^The mixture yields (**9a** + **7a**) were obtained after work-up. ^c^The ratio of **9a/7a** was determined by ^1^H NMR.

Encouraged by the achievements on catalytic asymmetric 1,3-dipolar cycloaddition to build octahydropyrrolo[3,4-*c*]pyrrole frameworks [7,9], we screened the optimal reaction conditions for preparing the racemic pyrrolidine **11a** using *N*-methylmaleimide (**10a**) as the simple dipolarophile without any chiral catalysts/ligands. To our delight, in the presence of 10 mol % of AgOAc, the reaction reached almost completion in CH_2_Cl_2_ within 1 h at room temperature and afforded the desired cycloadduct in moderate yield (64%, Table 2, entry 1). The annulation process did not occur without the catalyst (Table 2, entry 2). Since most of the 1,3-dipolar cycloadditions are known to work best in nonpolar solvents, toluene instead of CH_2_Cl_2_ was selected as the reaction solvent. Unfortunately, toluene was not a good choice even the reaction time was prolonged to 2 h (36% yield, Table 2, entry 3). Switching the base Et_3_N to DBU resulted in a significant decrease of product yield (Table 2, entry 4). Given that the crude azomethine ylide **9a** was used without any purification, and **9a** might be sensitive to other decomposition pathways, we increased the amount of **9a** up to 1.5 equivalents, which delivered the bicyclic pyrrolidine **11a** in excellent yield (82%, Table 2, entry 5). Prolonging the reaction time from 3 h to 6 h had no influence on the yield (Table 2, entry 6), which demonstrated that appropriate increase on the ratio of **9a**/**10a** was beneficial to the completion of this cycloaddition. Finally, the use of Et_3_N as the base and CH_2_Cl_2_ as the solvent, and the suitable ratio of **9a**/**10a** at 1.5:1 catalyzed by 10 mol % of AgOAc were identified as the optimal reaction conditions.

**Table 2:** Optimization of the reaction conditions of the cycloaddition reaction^a^.

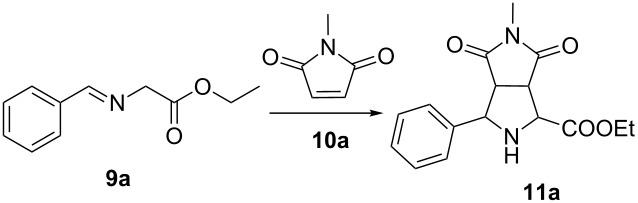					

entry	base	catalyst	solvent	time	yield (%)^b^

1	Et_3_N	AgOAc	CH_2_Cl_2_	1 h	64
2	Et_3_N	–	CH_2_Cl_2_	24 h	trace
3	Et_3_N	AgOAc	toluene	2 h	36
4	DBU	AgOAc	CH_2_Cl_2_	1 h	39
5^c^	Et_3_N	AgOAc	CH_2_Cl_2_	3 h	82
6^c^	Et_3_N	AgOAc	CH_2_Cl_2_	6 h	80

^a^General reaction conditions: **9a** (2.5 mmol), **10a** (2.5 mmol), AgOAc (0.25 mmol) and base (0.25 mmol), rt. ^b^Isolated yield. ^c^**9a/10a**/AgOAC/Et_3_N = 1.5:1:0.1:0.1.

Finally, the transformation of the cycloadduct **11a** to pyrrolo[3,4-*c*]pyrrole-1,3-dione **12a** was investigated (Table 3). For the operational simplicity and continuity of this one-pot condensation–cycloaddition–aromatization reaction, CH_2_Cl_2_ was firstly selected as solvent instead of the generally used toluene [10,25–26] for this oxidation step. Unfortunately, the desired pyrrole product **12a** was obtained only in 41% yield with DDQ (4 equiv) as oxidant at room temperature for 48 h (Table 3, entry 1). As expected, toluene as solvent improved the reaction outcome to afford **12a** in a good yield up to 71% (Table 3, entry 2). Subsequently, reducing the amount of DDQ (2 equiv) led to a significantly decreased yield (Table 3, entry 3), and also the addition of silica gel [9] as an additive had no considerable effect on the yield (Table 3, entry 4 vs entry 2). The results demonstrated that using 4 equiv DDQ in toluene were the best conditions for this oxidation protocol.

**Table 3:** Optimization of the reaction conditions of the oxidation reaction.

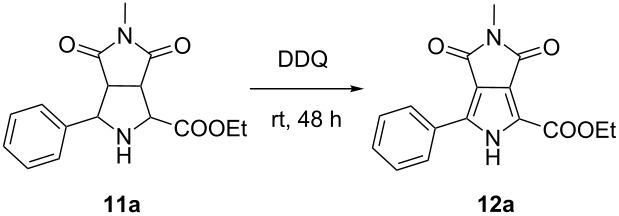				

entry	catalyst	solvent	ratio of **11a**/DDQ	yield (%)^a^

1	–	CH_2_Cl_2_	1:4	41
2	–	toluene	1:4	71
3	–	toluene	1:2	43
4	silica gel^b^	toluene	1:4	68

^a^Isolated yield. ^b^10% Amount of **11a**.

**Scheme 2:**
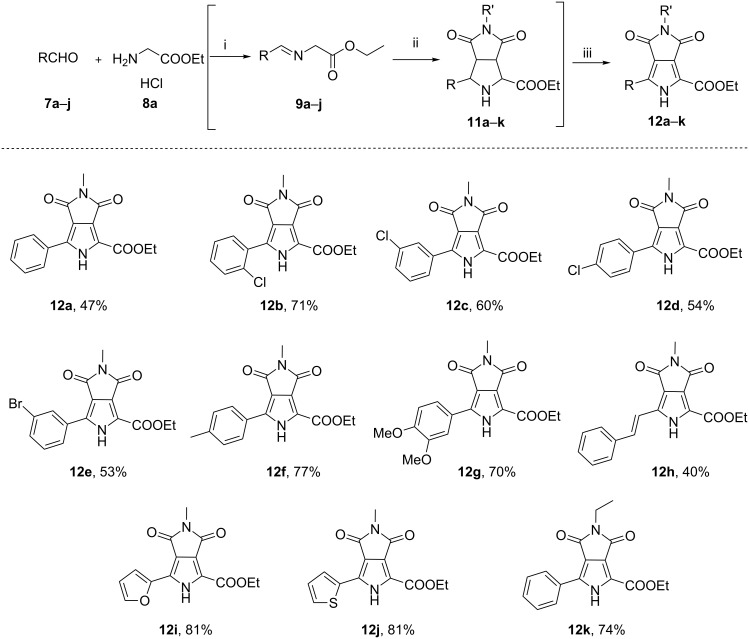
Scope of one-pot synthesis of pyrrolo[3,4-*c*]pyrrole-1,3-diones **12a**–**k**. General reaction conditions: (i) aldehydes **7a**–**j** (3 mmol), ethyl glycinate hydrochloride (**8a**, 4.5 mmol), Et_3_N, MgSO_4_, CH_2_Cl_2_, rt, 8 h; (ii) *N*-methylmaleimide (**10a**, 2 mmol) or *N*-ethylmaleimide (**10b**, 2 mmol), AgOAc (0.2 mmol), CH_2_Cl_2_, rt, 3 h; (iii) DDQ (8 mmol), toluene, rt, 12–80 h.

With the optimized reaction conditions for each step in hand, we prepared **12a** starting from benzaldehyde and ethyl glycinate hydrochloride through three steps but via a one-pot process without any purification for intermediates **9a** and **11a** (see Supporting Information File 1). To our delight, the reaction outcome was satisfactory with a total yield of 47% for three-steps which was equivalent to the stepwise reactions (Scheme 2, **12a**). In order to evaluate the generality of this process, we explored a variety of aldehydes **7b**–**j**, and the results are shown in Scheme 2. All tested benzaldehydes bearing electron-withdrawing or electron-donating substituents on the phenyl ring reacted smoothly via condensation–cycloaddition–oxidation in the one-pot process, delivering the corresponding pyrrolo[3,4-*c*]pyrrole-1,3-diones in moderate to good yields (53–77%). Heteroaromatic aldehydes **7i** and **7j** afforded the products **12i** and **12j** in excellent total yields of 81%. Additionally, cinnamaldehyde (**7h**) was also well tolerated, giving the product **12h** in moderate yield (40%). Finally, to further explore the scope and generality of this protocol, *N*-ethylmaleimide (**10b**) instead of *N*-methylmaleimide (**10a**) as another simple dipolarophile was also examined; the target product **12k** was prepared smoothly in high yield (74%). This concise and efficient protocol displays the potentiality for scale-up synthesis of pyrrolo[3,4-*c*]pyrrole-1,3-diones.

**Scheme 3:**
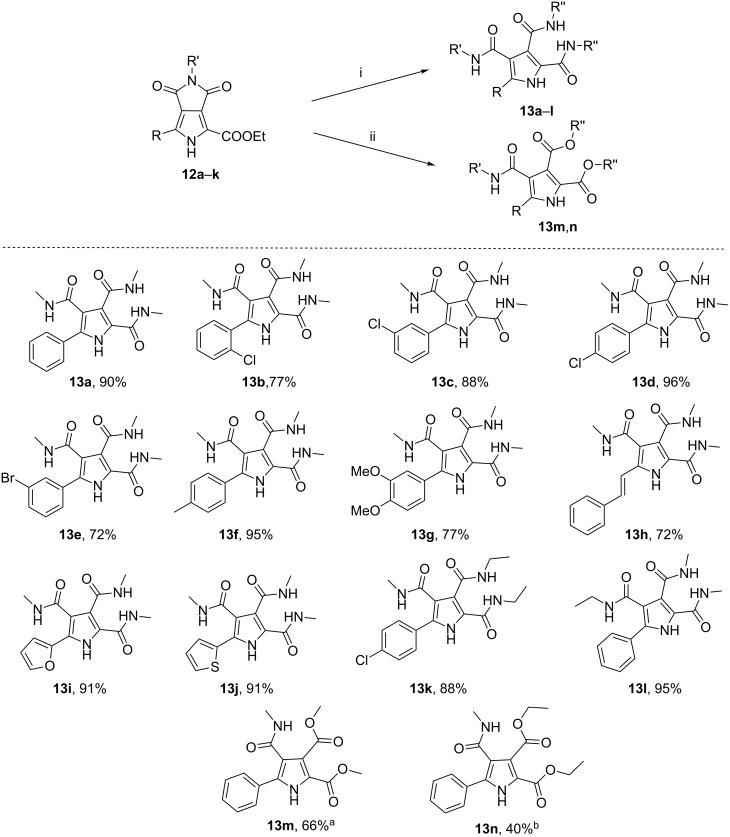
Scope of the synthesis of highly substituted pyrrole derivatives **13a**–**n**. General reaction conditions: (i) **12a**–**k** (0.17 mmol), methylamine or ethylamine solution in EtOH (3 mL), 80 °C, in a sealed tube for 3 h; (ii) ^a^**12a** (0.17 mmol), MeONa (3 equiv), MeOH, rt, 24 h; ^b^**12a** (0.17 mmol), EtONa (3 equiv), EtOH, rt, 36 h.

**Scheme 4:**
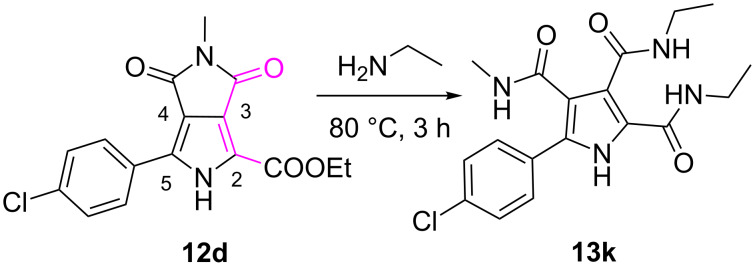
Synthesis of **13k** from **12d** and ethylamine.

Having the pyrrolo[3,4-*c*]pyrrole-1,3-diones with diversified substituents in hand, polysubstituted pyrrole derivatives with three amide groups **13a**–**l** were prepared smoothly by treating **12a**–**k** with methylamine or ethylamine in EtOH at 80 °C in good to excellent yields (72–96%, Scheme 3). The structures of highly substituted pyrrole derivatives prompted us to envisage the reasonable mechanism for the formation of 3,4-diamide groups, besides the amidation of ethyl ester group on the 2-position to give one amide group on the pyrrole ring. Due to the electron-withdrawing effect of the ester group at the 2-position in **12a**–**k**, the carbonyl group at the 3-position of the pyrrole was easily attacked by the nucleophilic amine (compared with the carbonyl at the 4-position, shown in Scheme 4) exemplified by the reaction of **12d** with ethylamine to afford **13k**. NOESY analysis of **13k** was used to confirm the relative positions of 3,4-diamide groups formed. NOE correlations between an H of the methylamide at the 4-position and H2 or H6 of the 4-chlorophenyl moiety supported the reaction result (see Supporting Information File 1). Additionally, the corresponding pyrroles with more functionalized substituents with both amide and ester group **13m**,**n** could also be readily achieved by treatment of **12a** with MeONa in MeOH or EtONa in EtOH at room temperature in moderate to good yields (40–66%, Scheme 3). It was noted that the ethyl ester in **12a** was replaced by methyl ester through transesterification when **12a** was treated with MeONa in MeOH to afford **13m**. Therefore, this efficient protocol developed could meet our requirement to basically construct a small library of highly functionalized pyrrole derivatives for antibacterial screening or for further chemical manipulations to get more promising compounds.

## Conclusion

In summary, we have developed an efficient, operationally simple protocol to afford pyrrolo[3,4-*c*]pyrrole-1,3-diones via 1,3-dipolar cycloaddition of azomethine ylides from diverse commercially available aldehydes in good to excellent yields and a facile access to highly functionalized pyrroles. Notably, the versatile conversion of the cycloaddition adducts leads to fascinating polysubstituted pyrrole compounds, which can deliver potentially valuable building blocks serving as precursors for drug discovery. Further research on the development of more diversified pyrrolo[3,4-*c*]pyrrole-1,3-diones and more complicated polysubstituted pyrroles is currently underway in our laboratory.

## Supporting Information

File 1 General information, experimental details, characterization data, copies of ^1^H and ^13^C NMR spectra of **12a**–**k** and **13a**–**n**, and NOESY spectra of compound **13k**.
