# Supplementary material for: An efficient and facile access to highly functionalized pyrrole derivatives
Source: Beilstein J Org Chem. 2018 Apr 20;14:884–90. doi: 10.3762/bjoc.14.75 (PMC5942380; doi:10.3762/bjoc.14.75)

Supporting Information

for

An efficient and facile access to highly functionalized pyrrole derivatives

Meng Gao, Wenting Zhao, Hongyi Zhao, Ziyun Lin, Dongfeng Zhang* and Haihong Huang*

Address: State Key Laboratory of Bioactive Substance and Function of Natural Medicines & Beijing Key Laboratory of Active Substance Discovery and Druggability Evaluation, Institute of Materia Medica, Peking Union Medical College and Chinese Academy of Medical Sciences, 1 Xian Nong Tan Street, Beijing 100050, China

Email: Dongfeng Zhang* - zdf@imm.ac.cn; Haihong Huang* - joyce@imm.ac.cn

* Corresponding author

General information, experimental details, characterization data, copies of ^{1}H and ^{13}C NMR spectra of 12a–k and 13a–n, and NOESY spectra of compound 13k

Table of Contents

General information **S2**

Typical procedure for the synthesis of products..**S2–S8**

Copies of ^{1}H and ^{13}C NMR spectra of products.......................................**S9–S33**

NOESY spectroscopy of compound 13k…..**S34**

General information

All the starting materials were obtained from commercial suppliers and were used without further purification unless stated otherwise. Reactions were monitored by TLC (silica gel GF254). Products were purified by column chromatography on silica gel (200–300 mesh). ^{1}H spectra were recorded on a Varian 400 NMR or 500 NMR spectrometer using $CDCl_3$, CD_3OD or DMSO-d_6 as a solvent and tetramethylsilane (TMS) as an internal standard. Chemical shift (δ) are reported in parts per million (ppm) and coupling constants (*J*) are reported in Hertz (Hz). Data are represented as follows: chemical shift, multiplicity (br = broad, s = singlet, d = doublet, t = triplet, q = quartet, m = multiplet), coupling constants in Hertz (Hz), integration. All melting points were measured with a microscope melting point apparatus (MP-J3, Yanaco) and were uncorrected. High-resolution mass spectra were determined on Thermo Exactive Orbitrap plus mass spectrometer.

Typical procedure for the synthesis of 12a–k

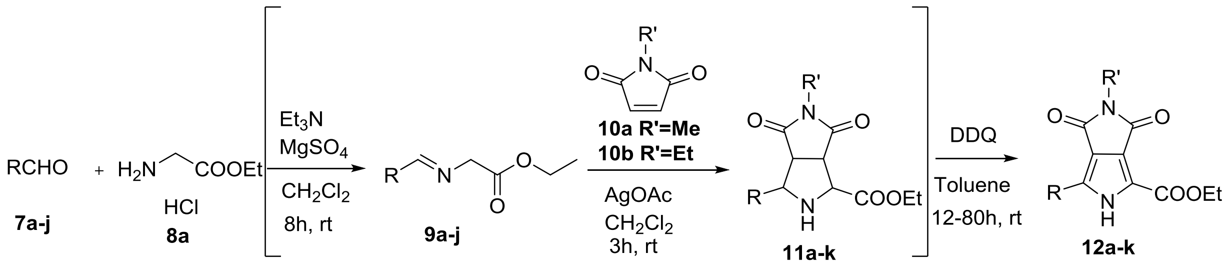

To a 25 mL flask were added aldehyde **7a–j** (3 mmol), ethyl glycinate hydrochloride **8a** (0.63 g, 4.5 mmol), Et_3N (0.62 mL, 4.5 mmol), $MgSO_4$ (0.54 g, 4.5 mmol) and CH_2Cl_2 (10 mL). The mixture was stirred vigorously at room temperature for 8 h. The mixture was filtered, the filtrate was added 20 mL water and extracted with CH_2Cl_2 (3 × 10 mL). The combined organic phase was washed with brine, dried over anhydrous Na_2SO_4, evaporated in vacuo to remaining about 10 mL. Then, to the residue were added maleimide (2 mmol), AgOAc (0.03 g, 0.2 mmol) and Et_3N (28 μL, 0.2 mmol), the mixture was stirred vigorously at room temperature for 3 h and filtered; the filtrate was evaporated in vacuo. Toluene (10 mL) and DDQ (1.82 g, 8 mmol) were added into the above resulted residue. The mixture was stirred vigorously at room temperature for 12–80 h and quenched with 20 mL saturated sodium bicarbonate solution, extracted with CH_2Cl_2 (6 × 20 mL). The combined organic phase was washed with brine, dried over anhydrous Na_2SO_4, and

evaporated in vacuo. The residue was purified by column chromatography on silica gel with petroleum/ethyl acetate (5:1~1:1) to give the target products **12a–k**.

Ethyl 5-methyl-4,6-dioxo-3-phenyl-2,4,5,6-tetrahydropyrrolo[3,4-*c*]pyrrole-1-carboxylate (12a). White solid, 47% yield, m.p.: 223-225℃. ^{1}H NMR (400 MHz, $CDCl_3$) δ 9.69 (s, 1H), 8.10-8.07 (m, 2H), 7.55-7.51 (m, 2H), 7.48-7.46 (m, 1H), 4.47 (q, *J*= 7.2 Hz, 2H), 3.14 (s, 3H), 1.47 (t, *J* = 7.2 Hz, 3H). ^{13}C NMR (100 MHz, DMSO-d_6) δ 164.3, 162.3, 159.0, 134.7, 130.2, 129.2, 128.6, 127.6, 125.0, 119.7, 118.5, 61.5, 24.5, 14.6. HR-MS (ESI): *m/z* $[M+H]^+$calcd for $C_{16}H_{15}N_2O_4$: 299.1026; found: 299.1018.

Ethyl 3-(2-chlorophenyl)-5-methyl-4,6-dioxo-2,4,5,6-tetrahydropyrrolo[3,4-*c*]pyrrole-1-carboxylate (12b). White solid, 71% yield, m.p.: 180-182℃. ^{1}H NMR (400 MHz, $CDCl_3$) δ 10.54 (s, 1H), 8.48 (dd, *J* = 8.0, 2.0 Hz, 1H), 7.51-7.44 (m, 2H), 7.41-7.36 (m, 1H), 4.47 (q, *J* = 7.2 Hz, 2H), 3.14 (s, 3H), 1.47 (t, *J* = 7.2 Hz, 3H). ^{13}C NMR (100 MHz, DMSO-d_6) δ 163.6, 162.5, 158.9, 133.2, 132.9, 131.9, 130.3, 130.2, 128.3, 127.6, 124.0, 120.9, 119.5, 61.6, 24.4, 14.7. HR-MS (ESI): *m/z* $[M+H]^+$calcd for $C_{16}H_{14}ClN_2O_4$: 333.0637; found: 333.0630.

Ethyl 3-(3-chlorophenyl)-5-methyl-4,6-dioxo-2,4,5,6-tetrahydropyrrolo[3,4-*c*]pyrrole-1-carboxylate (12c). White solid, 60% yield, m.p.: 200-202℃. ^{1}H NMR (400 MHz, $CDCl_3$) δ 9.99 (s, 1H), 8.11-8.10 (m, 1H), 8.04 (dt, *J* = 6.8, 2.0 Hz, 1H), 7.47-7.41 (m, 2H), 4.46 (q, *J* = 7.2 Hz, 2H), 3.15 (s, 3H), 1.47 (t, *J* = 7.2 Hz, 3H). ^{13}C NMR (100 MHz, DMSO-d_6) δ 164.2, 162.2, 158.9, 134.2, 132.8, 131.1, 130.6, 129.8, 127.0, 126.0, 124.9, 120.1, 119.3, 61.6, 24.5, 14.6. HR-MS (ESI): *m/z* $[M+H]^+$calcd for $C_{16}H_{14}ClN_2O_4$: 333.0637; found: 333.0630.

Ethyl 3-(4-chlorophenyl)-5-methyl-4,6-dioxo-2,4,5,6-tetrahydropyrrolo[3,4-*c*]pyrrole-1-carboxylate (12d). White solid, 54% yield, m.p.: >250℃. ^{1}H NMR (500 MHz, DMSO-d_6) δ 13.25 (s, 1H), 8.40 (d, *J* = 8.5 Hz, 2H), 7.63 (d, *J* = 8.0 Hz, 2H), 4.38 (q, *J* = 7.0 Hz, 2H), 2.99 (s, 3H), 1.38 (t, *J* = 7.0 Hz, 3H). ^{13}C NMR (100 MHz, DMSO-d_6) δ 164.2, 162.1, 159.0, 134.8, 133.3, 129.3, 129.2, 127.5, 124.9, 120.0, 118.9, 61.6, 24.5, 14.6. HR-MS (ESI): *m/z* $[M+H]^+$calcd for $C_{16}H_{14}ClN_2O_4$: 333.0637; found: 333.0633.

Ethyl 3-(3-bromophenyl)-5-methyl-4,6-dioxo-2,4,5,6-tetrahydropyrrolo[3,4-*c*]pyrrole-1-carboxylate (12e). White solid, 53% yield, m.p.: 218-220℃. ^{1}H NMR (500 MHz, $CDCl_3$) δ 9.82 (s, 1H), 8.24 (s, 1H), 8.07 (d, *J* = 8.0 Hz, 1H), 7.58 (d, *J* = 8.0 Hz, 1H), 7.41-7.38 (m, 1H), 4.47 (q, *J* = 7.0 Hz, 2H), 3.14 (s, 3H), 1.47 (t, *J* = 7.0 Hz, 3H). ^{13}C NMR (100 MHz, DMSO-d_6) δ 164.3,

162.2, 159.0, 132.8, 132.7, 131.4, 130.9, 129.9, 126.5, 125.0, 122.7, 120.2, 119.4, 61.7, 24.6, 14.7. HR-MS (ESI): *m/z* $[M+H]^+$calcd for $C_{16}H_{14}BrN_2O_4$: 377.0132; found: 377.0129.

Ethyl 5-methyl-4,6-dioxo-3-(*p*-tolyl)-2,4,5,6-tetrahydropyrrolo[3,4-*c*]pyrrole-1-carboxylate (12f). White solid, 77% yield, m.p.: 195-197℃. ^1H NMR (400 MHz, $CDCl_3$) δ 9.69 (s, 1H), 7.98 (d, *J* = 8.4 Hz, 2H), 7.32 (d, *J* = 8.4 Hz, 2H), 4.46 (q, *J* = 7.2 Hz, 2H), 3.13 (s, 3H), 2.42 (s, 3H), 1.47 (t, *J* = 7.2 Hz, 3H). ^{13}C NMR (100 MHz, DMSO-d_6) δ 164.3, 162.4, 159.1, 140.1, 135.0, 129.8, 127.6, 126.0, 125.0, 119.5, 118.0, 61.5, 24.5, 21.5, 14.6. HR-MS (ESI): *m/z* $[M+H]^+$calcd for $C_{17}H_{17}N_2O_4$: 313.1183; found: 313.1177.

Ethyl 3-(3,4-dimethoxyphenyl)-5-methyl-4,6-dioxo-2,4,5,6-tetrahydropyrrolo[3,4-*c*]pyrrole-1-carboxylate (12g). Yellow solid, 70% yield, m.p.: 240-242℃. ^1H NMR (400 MHz, $CDCl_3$) δ 9.64 (s, 1H), 8.05 (d, *J* = 2.0 Hz, 1H), 7.42 (dd, *J* = 8.4, 2.4 Hz, 1H), 6.95 (d, *J* = 8.4 Hz, 1H), 4.46 (q, *J* = 7.2 Hz, 2H), 4.04 (s, 3H), 3.95 (s, 3H), 3.14 (s, 3H), 1.48 (t, *J* = 7.2 Hz, 3H). ^{13}C NMR (100 MHz, DMSO-d_6) δ 164.5, 162.4, 159.2, 150.5, 149.2, 135.4, 124.9, 121.3, 120.8, 119.1, 117.3, 112.1, 110.9, 61.5, 56.1, 56.0, 24.5, 14.6. HR-MS (ESI): *m/z* $[M+H]^+$calcd for $C_{18}H_{19}N_2O_6$: 359.1238; found: 359.1228.

Ethyl (*E*)-5-methyl-4,6-dioxo-3-styryl-2,4,5,6-tetrahydropyrrolo[3,4-*c*]pyrrole-1-carboxylate (12h). White solid, 40% yield, m.p.: 246-248℃. ^1H NMR (400 MHz, $CDCl_3$) δ 9.76 (s, 1H), 7.99 (d, *J* = 16.4 Hz, 1H), 7.58-7.56 (m, 2H), 7.42-7.35 (m, 3H), 6.97 (d, *J* = 16.4 Hz, 1H), 4.46 (q, *J* = 7.2 Hz, 2H), 3.13 (s, 3H), 1.47 (t, *J* = 7.2 Hz, 3H). ^{13}C NMR (100 MHz, DMSO-d_6) δ 164.4, 162.6, 159.0, 136.4, 136.3, 133.9, 129.7, 129.6, 127.3, 124.8, 120.0, 118.7, 118.5, 115.9, 61.6, 24.5, 14.7. HR-MS (ESI): *m/z* $[M+H]^+$calcd for $C_{18}H_{17}N_2O_4$: 325.1183; found: 325.1175.

Ethyl 3-(furan-2-yl)-5-methyl-4,6-dioxo-2,4,5,6-tetrahydropyrrolo[3,4-*c*]pyrrole-1-carboxylate (12i). White solid, 81% yield, m.p.: 249-251℃. ^1H NMR (400 MHz, $CDCl_3$) δ 9.70 (s, 1H), 7.53 (dd, *J* = 2.0, 0.8 Hz, 1H), 7.44 (dd, *J* = 3.6, 0.8 Hz, 1H), 6.60 (dd, *J* = 3.6, 2.0 Hz, 1H), 4.46 (q, *J* = 7.2 Hz, 2H), 3.12 (s, 3H), 1.47 (t, *J* = 7.2 Hz, 3H). ^{13}C NMR (100 MHz, DMSO-d_6) δ 163.2, 162.3, 158.9, 145.2, 144.2, 124.6, 124.2, 119.4, 117.0, 112.9, 112.3, 61.5, 24.4, 14.6. HR-MS (ESI): *m/z* $[M+H]^+$calcd for $C_{14}H_{13}N_2O_5$: 289.0819; found: 289.0810.

Ethyl 5-methyl-4,6-dioxo-3-(thiophen-2-yl)-2,4,5,6-tetrahydropyrrolo[3,4-*c*]pyrrole-1-carboxylate (12j). White solid, 81% yield, m.p.: 228-230℃. ^1H NMR (400 MHz, $CDCl_3$) δ 9.58 (s, 1H), 8.03 (dd, *J* = 3.6, 1.2 Hz, 1H), 7.45 (dd, *J* = 5.2, 1.2 Hz, 1H), 7.17 (dd, *J* = 5.2, 3.6 Hz, 1H),

4.46 (q, J = 7.2 Hz, 2H), 3.13 (s, 3H), 1.46 (t, J = 7.2 Hz, 3H). ^{13}C NMR (100 MHz, DMSO-d_6) δ 163.7, 162.3, 159.1, 130.9, 129.8, 129.3, 128.5, 128.4, 124.6, 119.4, 117.4, 61.6, 24.5, 14.6. HR-MS (ESI): m/z [M+H]$^+$calcd for $C_{14}H_{13}N_2O_4S$: 305.0591; found: 305.0581.

Ethyl 5-ethyl-4,6-dioxo-3-phenyl-2,4,5,6-tetrahydropyrrolo[3,4-*c*]pyrrole-1-carboxylate (12k). White solid, 74% yield, m.p.: 214-215℃. ^{1}H NMR (400 MHz, $CDCl_3$) δ 9.77 (s, 1H), 8.11 -8.08(m, 2H), 7.55-7.46 (m, 3H), 4.47 (q, J = 7.2 Hz, 2H), 3.71 (q, J = 7.2 Hz, 2H), 1.48 (t, J = 7.2 Hz, 3H), 1.26 (t, J = 7.2 Hz, 3H). ^{13}C NMR (100 MHz, DMSO-d_6) δ 163.9, 162.0, 159.1, 134.8, 130.2, 129.2, 128.6, 127.7, 124.9, 119.8, 118.5, 61.5, 32.9, 14.6, 14.3. HR-MS (ESI): m/z [M+H]$^+$ calcd for $C_{17}H_{17}N_2O_4$: 313.1183; found: 313.1175.

Typical procedure for the synthesis of highly substituted pyrroles 13a–n

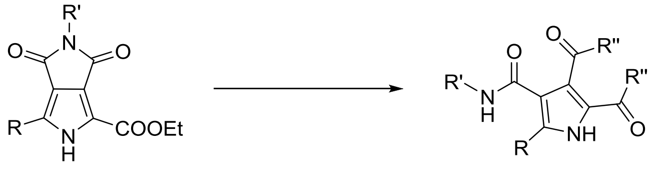

12a–k (0.167 mmol) and methylamine or ethylamine alcohol solution (3 mL) were stirred at 80 °C for 3 h in a 10 mL sealed tube. Then the mixture was evaporated in vacuo and purified by column chromatography on silica gel with 3% MeOH/CH_2Cl_2 to afford the target compounds **13a–l**.

12a (50 mg, 0.167 mmol) and sodium methoxide (27 mg, 0.501 mmol) were added into methanol (5 mL) and stirred at room temperature for 24 h. Then the mixture was diluted with 1 M HCl (10 mL) and extracted with CH_2Cl_2 (3 × 15 mL). The combined organic phase was washed with brine, dried over anhydrous Na_2SO_4, evaporated in vacuo. The residue was purified by column chromatography on silica gel with 8% MeOH/ CH_2Cl_2 to give **13m** (35 mg, 66%) as a white solid.

13n was synthesized by the general procedure as described in **13m**.

***N*^{2},*N*^{3},*N*^{4}-Trimethyl-5-phenyl-1*H*-pyrrole-2,3,4-tricarboxamide (13a).** White solid, 90% yield, m.p. >250℃. ^{1}H NMR (400 MHz, DMSO-d_6) δ 12.12 (s, 1H), 10.20 (d, J = 4.8 Hz, 1H), 8.77 (d, J = 4.4 Hz, 1H), 8.13 (d, J = 4.4 Hz, 1H), 7.52-7.50 (m, 2H), 7.42-7.32 (m, 3H), 2.83 (d, J = 4.4 Hz, 3H), 2.78 (d, J = 4.4 Hz, 3H), 2.58 (d, J = 4.8 Hz, 3H). ^{13}C NMR (100 MHz, DMSO-d_6) δ 167.9,

166.0, 160.8, 132.4, 130.7, 128.7, 128.4, 128.3, 127.9, 118.8, 118.0, 26.8, 26.6, 26.2. HR-MS (ESI): *m/z* $[M+H]^+$ calcd for $C_{16}H_{19}N_4O_3$: 315.1452; found: 315.1441.

5-(2-Chlorophenyl)-N^2,N^3,N^4-trimethyl-1*H*-pyrrole-2,3,4-tricarboxamide (13b). White solid, 77% yield, m.p.: 236-238℃. ^{1}H NMR (400 MHz, CD_3OD) δ 7.55-7.53 (m, 1H), 7.49-7.41 (m, 3H), 2.94 (s, 3H), 2.92 (s, 3H), 2.66 (s, 3H). ^{13}C NMR (100 MHz, DMSO-d_6) δ 166.8, 166.3, 160.6, 133.8, 133.4, 130.7, 130.6, 130.3, 129.7, 128.2, 127.0, 119.7, 117.3, 26.7, 26.6, 26.2. HR-MS (ESI): *m/z* $[M+H]^+$ calcd for $C_{16}H_{18}ClN_4O_3$: 349.1062; found: 349.1057.

5-(3-Chlorophenyl)-N^2,N^3,N^4-trimethyl-1*H*-pyrrole-2,3,4-tricarboxamide (13c). White solid, 88% yield, m.p. >250℃. ^{1}H NMR (500 MHz, DMSO-d_6) δ 12.30 (s, 1H), 10.16 (d, *J* = 4.0 Hz, 1H), 8.75 (d, *J* = 4.0 Hz, 1H), 8.21 (d, *J* = 4.5 Hz, 1H), 7.62 (s, 1H), 7.43-7.41 (m, 3H), 2.83 (d, *J* = 4.5 Hz, 3H), 2.78 (d, *J* = 4.5 Hz, 3H), 2.60 (d, *J* = 4.0 Hz, 3H). ^{13}C NMR (400 MHz, DMSO-d_6) δ 167.6, 165.8, 160.7, 133.4, 132.7, 130.7, 130.5, 128.4, 128.2, 128.1, 126.8, 119.4, 118.1, 26.8, 26.7, 26.2. HR-MS (ESI): *m/z* $[M+H]^+$ calcd for $C_{16}H_{18}ClN_4O_3$: 349.1062; found: 349.1053.

5-(4-Chlorophenyl)-N^2,N^3,N^4-trimethyl-1*H*-pyrrole-2,3,4-tricarboxamide (13d). White solid, 96% yield, m.p. >250℃. ^{1}H NMR (400 MHz, DMSO-d_6) δ 12.23 (s, 1H), 10.20 (s, 1H), 8.75 (s, 1H), 8.17 (d, *J* = 4.4 Hz, 1H), 7.51-7.46 (m, 4H), 2.83 (d, *J* = 4.8 Hz, 3H), 2.78 (d, *J* = 4.8 Hz, 3H), 2.59 (d, *J* = 4.4 Hz, 3H). ^{13}C NMR (100 MHz, DMSO-d_6) δ 167.7, 165.9, 160.7, 133.0, 131.2, 130.1, 129.6, 128.7, 128.3, 119.1, 118.0, 26.8, 26.7, 26.2. HR-MS (ESI): *m/z* $[M+H]^+$ calcd for $C_{16}H_{18}ClN_4O_3$: 349.1062; found: 349.1056.

5-(3-Bromophenyl)-N^2,N^3,N^4-trimethyl-1*H*-pyrrole-2,3,4-tricarboxamide (13e). White solid, 72% yield, m.p. >250℃. ^{1}H NMR (400 MHz, DMSO-d_6) δ 12.31 (s, 1H), 10.16 (d, *J* = 4.4 Hz, 1H), 8.75 (d, *J* = 4.8 Hz, 1H), 8.21 (d, *J* = 4.8 Hz, 1H), 7.76 (t, *J* = 1.6 Hz, 1H), 7.55-7.52 (m, 1H), 7.45-7.42 (m, 1H), 7.36 (t, *J* = 8.0 Hz, 1H), 2.83 (d, *J* = 4.4 Hz, 3H), 2.78 (d, *J* = 4.4 Hz, 3H), 2.60 (d, *J* = 4.8 Hz, 3H). ^{13}C NMR (100 MHz, DMSO-d_6) δ 167.6, 165.8, 160.6, 132.9, 131.1, 131.0, 130.7, 130.6, 128.4, 127.2, 121.9, 119.4, 118.1, 26.8, 26.7, 26.2. HR-MS (ESI): *m/z* $[M+H]^+$ calcd for $C_{16}H_{18}BrN_4O_3$: 393.0557; found: 393.0547.

N^2,N^3,N^4-Trimethyl-5-(*p*-tolyl)-1*H*-pyrrole-2,3,4-tricarboxamide (13f). White solid, 95% yield, m.p.: >250℃. ^{1}H NMR (500 MHz, DMSO-d_6) δ 12.04 (s, 1H), 10.21 (d, *J* = 4.0 Hz, 1H), 8.76 (d, *J* = 4.0 Hz, 1H), 8.10 (d, *J* = 4.5 Hz, 1H), 7.40 (d, *J* = 8.0 Hz, 2H), 7.21 (d, *J* = 7.5 Hz, 2H), 2.83 (d, *J* = 4.5 Hz, 3H), 2.78 (d, *J* = 4.5 Hz, 3H), 2.58 (d, *J* = 4.0 Hz, 3H), 2.32 (s, 3H). ^{13}C NMR (100

MHz, DMSO-d_6) δ 168.0, 166.0, 160.8, 137.8, 132.5, 129.2, 128.2, 127.9, 127.7, 118.4, 117.9, 26.8, 26.6, 26.2, 21.3. HR-MS (ESI): *m/z* $[M+H]^+$ calcd for $C_{17}H_{21}N_4O_3$: 329.1608; found: 329.1601.

5-(3,4-Dimethoxyphenyl)-*N*^{2},*N*^{3},*N*^{4}-trimethyl-1*H*-pyrrole-2,3,4-tricarboxamide (13g). White solid, 77% yield, m.p.: 236-238℃. ^{1}H NMR (500 MHz, DMSO-d_6) δ 12.05 (s, 1H), 10.25 (d, *J* = 4.5 Hz, 1H), 8.71 (d, *J* = 4.5 Hz, 1H), 8.14 (d, *J* = 4.0 Hz, 1H), 7.21 (s, 1H), 7.01-6.97 (m, 2H), 3.79 (s, 3H), 3.77 (s, 3H)，2.83 (d, *J* = 4.5Hz, 3H), 2.78 (d, *J* = 4.0 Hz, 3H), 2.60 (d, *J* = 4.5 Hz, 3H). ^{13}C NMR (100 MHz, DMSO-d_6) δ 168.2, 166.1, 160.8, 149.1, 148.7, 132.4, 127.5, 123.3, 120.6, 118.0, 117.8, 112.2, 111.9, 56.0, 55.9, 26.9, 26.7, 26.2. HR-MS (ESI): *m/z* $[M+H]^+$ calcd for $C_{18}H_{23}N_4O_5$: 375.1663; found: 375.1653.

(*E*)-*N*^{2},*N*^{3},*N*^{4}-Trimethyl-5-styryl-1*H*-pyrrole-2,3,4-tricarboxamide (13h). Yellow solid, 72% yield, m.p. >250℃. ^{1}H NMR (400 MHz, DMSO-d_6) δ 12.10 (s, 1H), 10.18 (d, *J* = 4.8 Hz, 1H), 8.94 (d, *J* = 4.8 Hz, 1H), 8.50 (d, *J* = 4.4 Hz, 1H), 7.50-7.45 (m, 3H), 7.39 (t, *J* = 7.6 Hz, 2H), 7.28 (t, *J* = 7.2 Hz, 1H), 7.11 (d, *J* = 16.8 Hz, 1H), 2.83 (d, *J* = 4.8 Hz, 3H), 2.80 (d, *J* = 4.8 Hz, 3H), 2.77 (d, *J* = 4.4 Hz, 3H). ^{13}C NMR (100 MHz, DMSO-d_6) δ 166.8, 166.2, 160.6, 137.3, 131.3, 130.0, 129.3, 128.5, 128.3, 126.7, 119.6, 118.0, 116.7, 26.9, 26.7, 26.2. HR-MS (ESI): *m/z* $[M+H]^+$ calcd for $C_{18}H_{21}N_4O_3$: 341.1608; found: 341.1600.

5-(Furan-2-yl)-*N*^{2},*N*^{3},*N*^{4}-trimethyl-1*H*-pyrrole-2,3,4-tricarboxamide (13i). White solid, 91% yield, m.p.: 238-240℃. ^{1}H NMR (400 MHz, DMSO-d_6) δ 12.18 (s, 1H), 10.15 (d, *J* = 4.8 Hz, 1H), 8.68 (d, *J* = 4.8 Hz, 1H), 8.35 (d, *J* = 4.8 Hz, 1H), 7.72-7.71 (m, 1H), 6.98-6.97 (m, 1H), 6.57-6.56 (m, 1H), 2.82 (d, *J* = 4.4 Hz, 3H), 2.77 (d, *J* = 4.8 Hz, 3H), 2.72 (d, *J* = 4.8 Hz, 3H). ^{13}C NMR (100 MHz, DMSO-d_6) δ 167.0, 165.8, 160.5, 145.4, 143.5, 127.7, 123.3, 118.1, 118.0, 112.1, 108.8, 26.7, 26.6, 26.2. HR-MS (ESI): *m/z* $[M+H]^+$ calcd for $C_{14}H_{17}N_4O_4$: 305.1244; found: 305.1236.

***N*^{2},*N*^{3},*N*^{4}-Trimethyl-5-(thiophen-2-yl)-1*H*-pyrrole-2,3,4-tricarboxamide (13j).** White solid, 91% yield, m.p.: 230-232℃. ^{1}H NMR (500 MHz, DMSO-d_6) δ 12.17 (s, 1H), 10.06 (d, *J* = 3.5 Hz, 1H), 8.53 (d, *J* = 3.5 Hz, 1H), 8.40 (d, *J* = 4.0 Hz, 1H), 7.58-7.55 (m, 2H), 7.10-7.08 (m, 1H), 2.82 (d, *J* = 4.0 Hz, 3H), 2.77 (d, *J* = 4.5 Hz, 3H), 2.68 (d, *J* = 4.0 Hz, 3H). ^{13}C NMR (125 MHz, DMSO-d_6) δ 167.0, 165.8, 160.6, 133.0, 127.7, 127.5, 127.3, 127.2, 126.2, 119.0, 118.0, 26.9, 26.7, 26.2. HR-MS (ESI): *m/z* $[M+H]^+$ calcd for $C_{14}H_{17}N_4O_3S$: 321.1016; found: 321.1008.

5-(4-Chlorophenyl)-*N*^{2},*N*^{3}-diethyl-*N*^{4}-methyl-1*H*-pyrrole-2,3,4-tricarboxamide (13k). White solid, 88% yield, m.p. >250℃. ^{1}H NMR (500 MHz, DMSO-d_6) δ 12.23 (s, 1H), 10.24 (t, J = 4.5 Hz, 1H), 8.68 (t, J = 4.5 Hz, 1H), 8.19 (d, J = 4.5 Hz, 1H), 7.51 (d, J = 8.5 Hz, 2H), 7.47 (d, J = 8.5 Hz, 2H), 3.29-3.25 (m, 4H), 2.60 (d, J = 4.5 Hz, 3H), 1.14 (t, J = 7.5 Hz, 3H), 1.09 (t, J = 7.5 Hz, 3H). ^{13}C NMR (100 MHz, DMSO-d_6) δ 167.8, 165.3, 160.0, 133.1, 131.1, 130.1, 129.7, 128.7, 128.4, 119.3, 118.1, 34.6, 34.2, 26.9, 15.2, 14.9. HR-MS (ESI): *m/z* [M+H]$^+$ calcd for $C_{18}H_{22}ClN_4O_3$: 377.1375; found: 377.1365.

***N*^{4}-Ethyl-*N*^{2},*N*^{3}-dimethyl-5-phenyl-1*H*-pyrrole-2,3,4-tricarboxamide (13l).** White solid, 95% yield, m.p.: 243-245℃. ^{1}H NMR (400 MHz, DMSO-d_6) δ 12.13 (s, 1H), 10.23-10.22 (m, 1H), 8.72-8.70 (m, 1H), 8.22-8.19 (m, 1H), 7.54-7.52 (m, 2H), 7.41-7.32 (m, 3H), 3.12-3.09 (m, 2H), 2.83 (d, J = 4.8 Hz, 3H), 2.78 (d, J = 4.4 Hz, 3H), 0.89 (t, J = 7.2 Hz, 3H). ^{13}C NMR (100 MHz, DMSO-d_6) δ 166.7, 165.7, 160.3, 131.9, 130.3, 128.3, 128.2, 128.0, 127.5, 118.6, 117.5, 34.1, 26.2, 25. 8, 13.9. HR-MS (ESI): *m/z* [M+H]$^+$ calcd for $C_{17}H_{21}N_4O_3$: 329.1608; found: 329.1597.

Dimethyl 4-(methylcarbamoyl)-5-phenyl-1*H*-pyrrole-2,3-dicarboxylate (13m). White solid, 66% yield, m.p.: 202-203℃. ^{1}H NMR (400 MHz, DMSO-d_6) δ 12.57 (s, 1H), 8.04 (d, J = 4.8 Hz, 1H), 7.51-7.49 (m, 2H), 7.45-7.42 (m, 3H), 3.76 (s, 3H), 3.56 (s, 3H), 2.72 (d, J = 4.8 Hz, 3H). ^{13}C NMR (100 MHz, DMSO-d_6) δ 165.0, 164.1, 160.4, 139.4, 130.7, 130.0, 129.6, 129.2, 128.2, 119.4, 112.5, 52.1, 51.5, 26.5. HR-MS (ESI): *m/z* [M+H]$^+$ calcd for $C_{16}H_{17}N_2O_5$: 317.1132; found: 317.1131.

Diethyl 4-(methylcarbamoyl)-5-phenyl-1*H*-pyrrole-2,3-dicarboxylate (13n). White solid, 40% yield, m.p.: 175-176℃. ^{1}H NMR (400 MHz, DMSO-d_6) δ 12.53 (s, 1H), 8.03 (d, J = 4.8 Hz, 1H), 7.51-7.49(m, 2H), 7.44-7.42 (m, 3H), 4.21 (q, J = 7.2 Hz, 2H), 4.02 (q, J = 7.2 Hz, 2H), 2.70 (d, J = 4.4 Hz, 3H), 1.24 (t, J = 7.2 Hz, 3H), 1.08 (t, J = 7.2 Hz, 3H). ^{13}C NMR (100 MHz, DMSO-d_6) δ 165.1, 163.5, 160.1, 139.5, 130.8, 130.2, 129.6, 129.1, 128.2, 119.6, 112.5, 60.6, 59.9, 26.4, 14.5, 14.2. HR-MS (ESI): *m/z* [M+H]$^+$ calcd for $C_{18}H_{21}N_2O_5$: 345.1445; found: 345.1447.

Copies of ^{1}H and ^{13}C NMR spectra

Ethyl 5-methyl-4,6-dioxo-3-phenyl-2,4,5,6-tetrahydropyrrolo[3,4-*c*]pyrrole-1-carboxylate (**12a**)

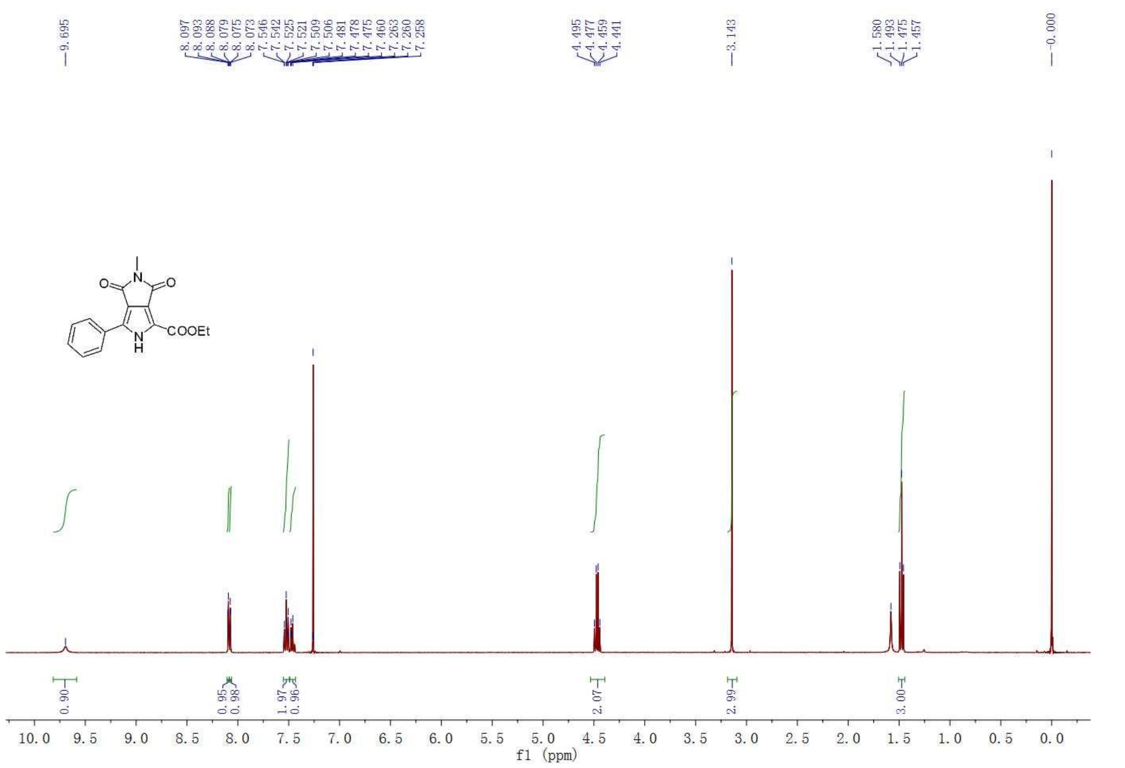

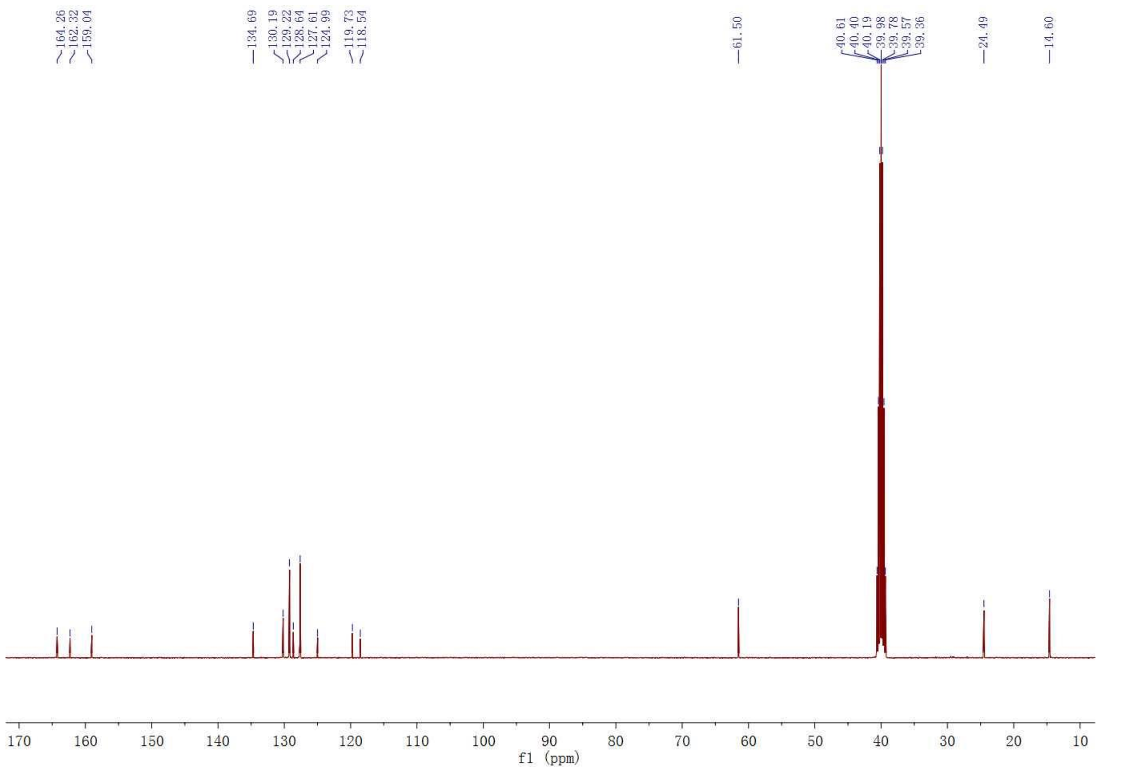

Ethyl 3-(2-chlorophenyl)-5-methyl-4,6-dioxo-2,4,5,6-tetrahydropyrrolo[3,4-*c*]pyrrole-1-carboxylate (**12b**)

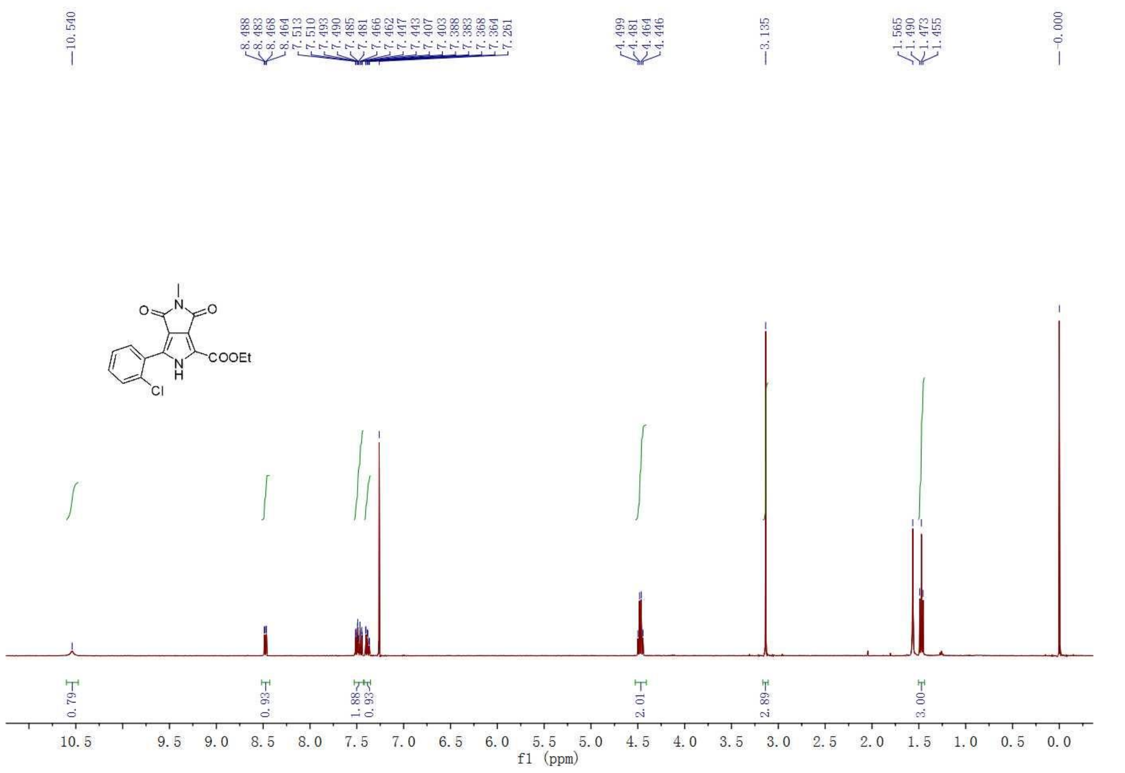

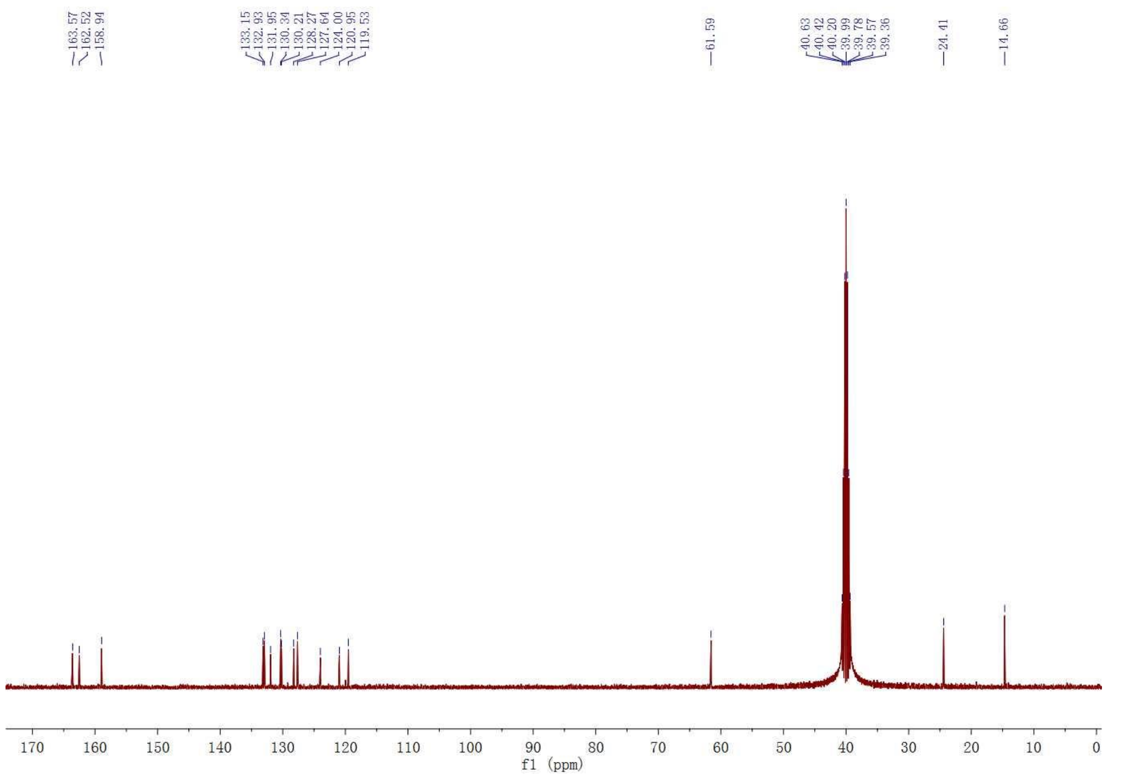

Ethyl 3-(3-chlorophenyl)-5-methyl-4,6-dioxo-2,4,5,6-tetrahydropyrrolo[3,4-*c*]pyrrole-1-carboxylate (**12c**)

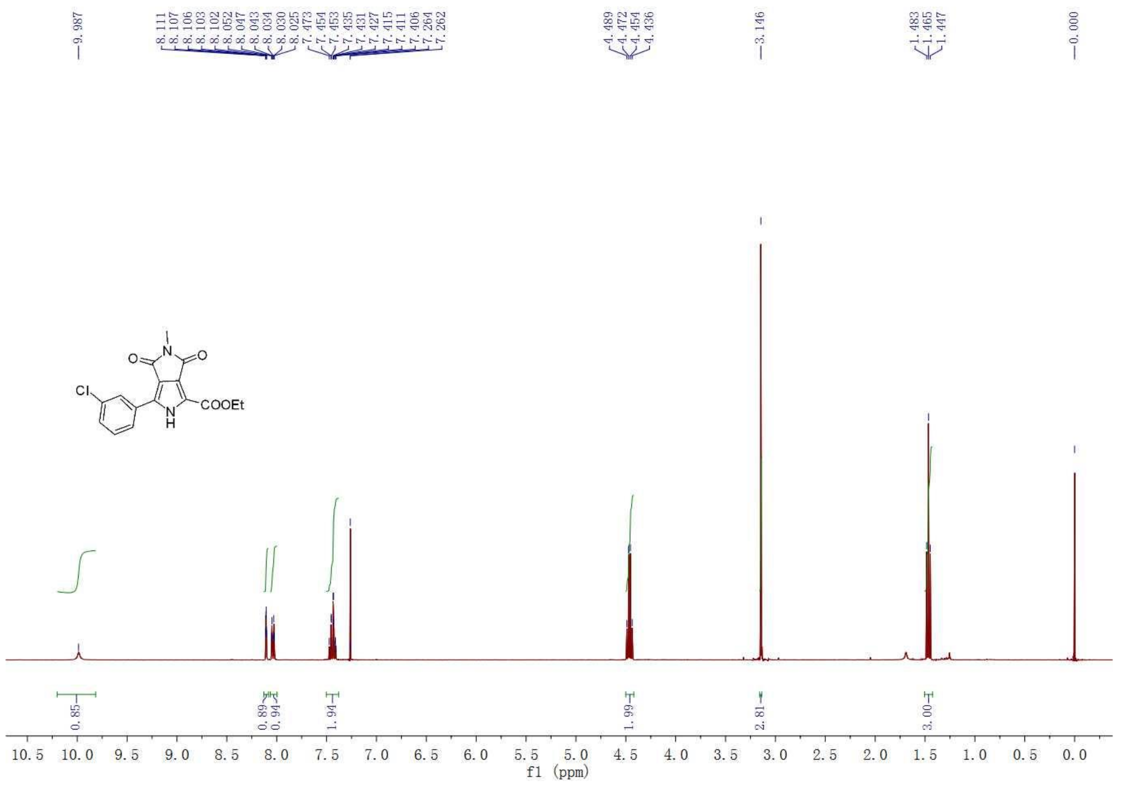

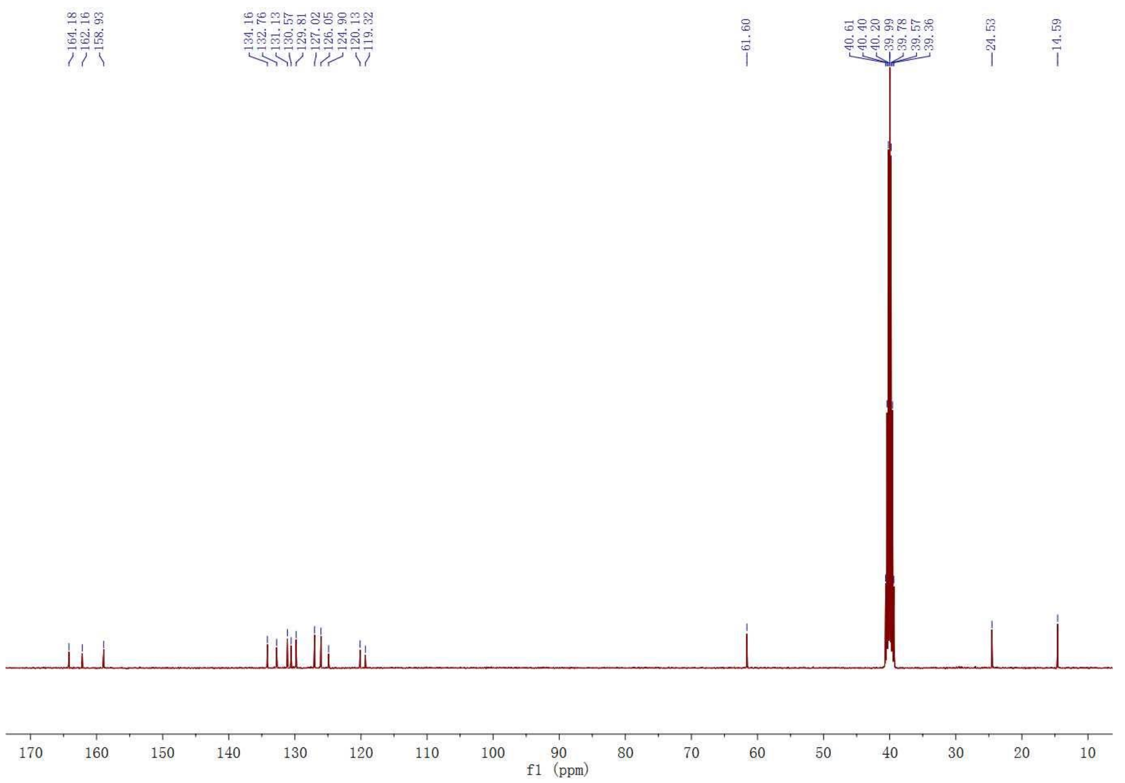

Ethyl 3-(4-chlorophenyl)-5-methyl-4,6-dioxo-2,4,5,6-tetrahydropyrrolo[3,4-*c*]pyrrole-1-carboxylate (**12d**)

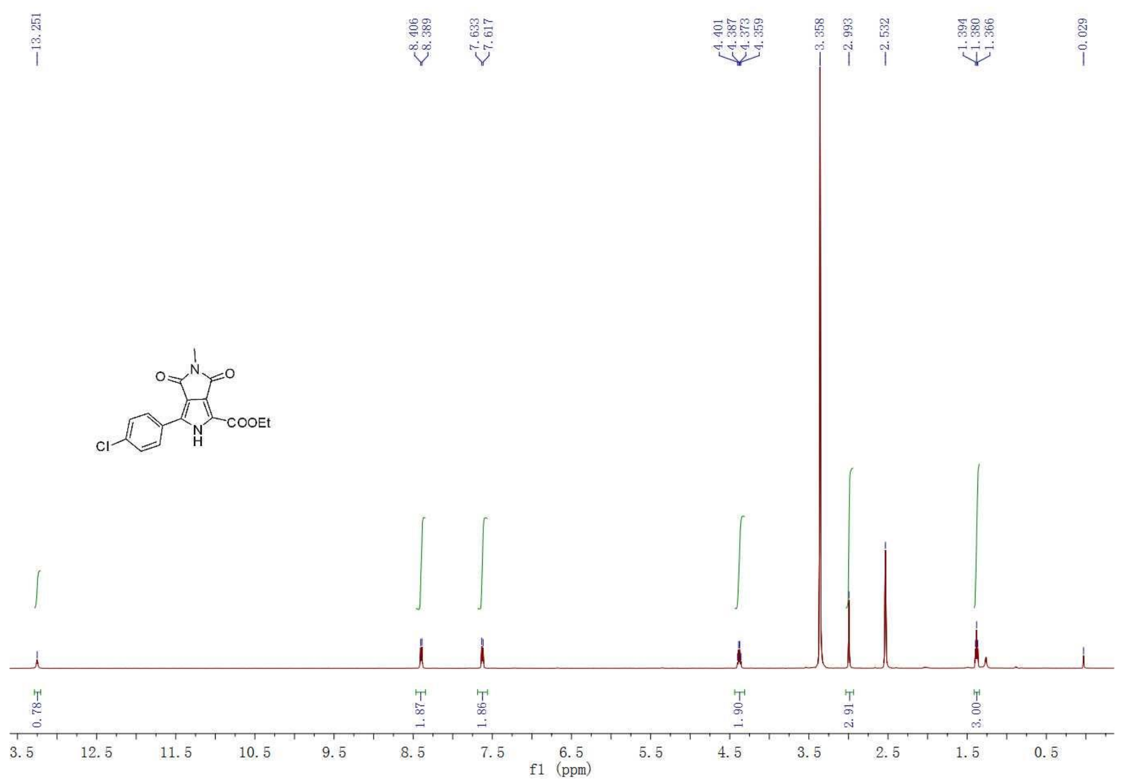

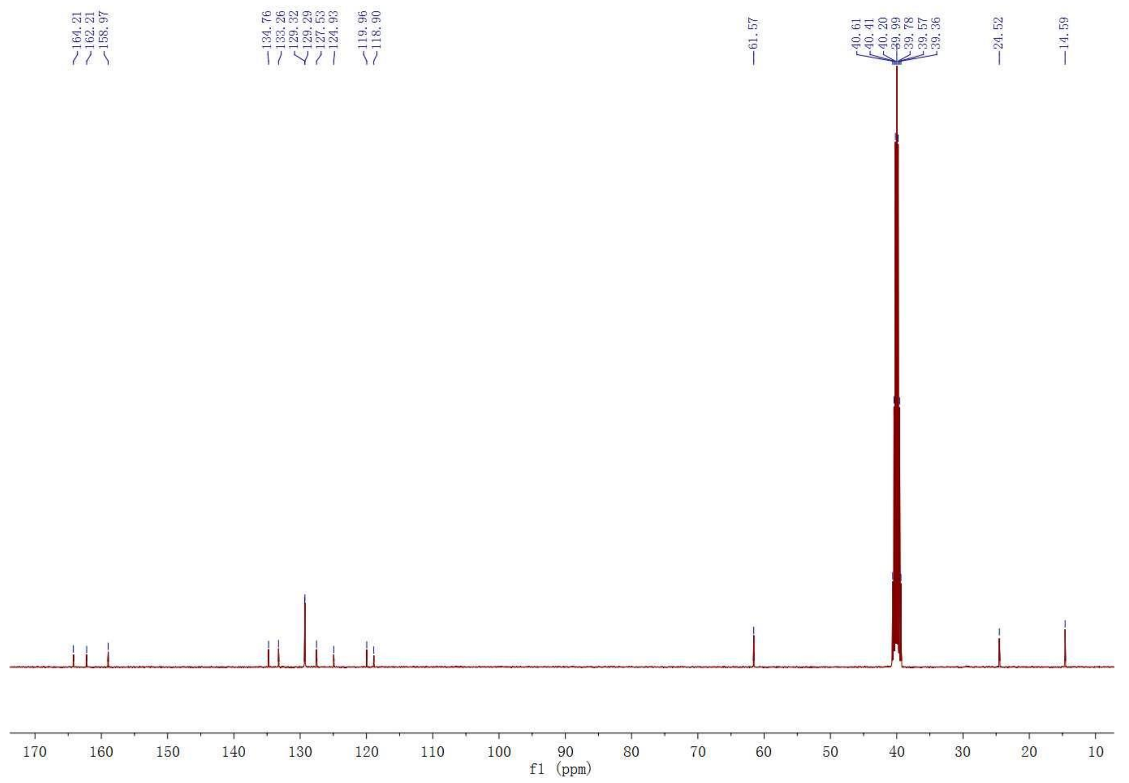

Ethyl 3-(3-bromophenyl)-5-methyl-4,6-dioxo-2,4,5,6-tetrahydropyrrolo[3,4-*c*]pyrrole-1-carboxylate (**12e**)

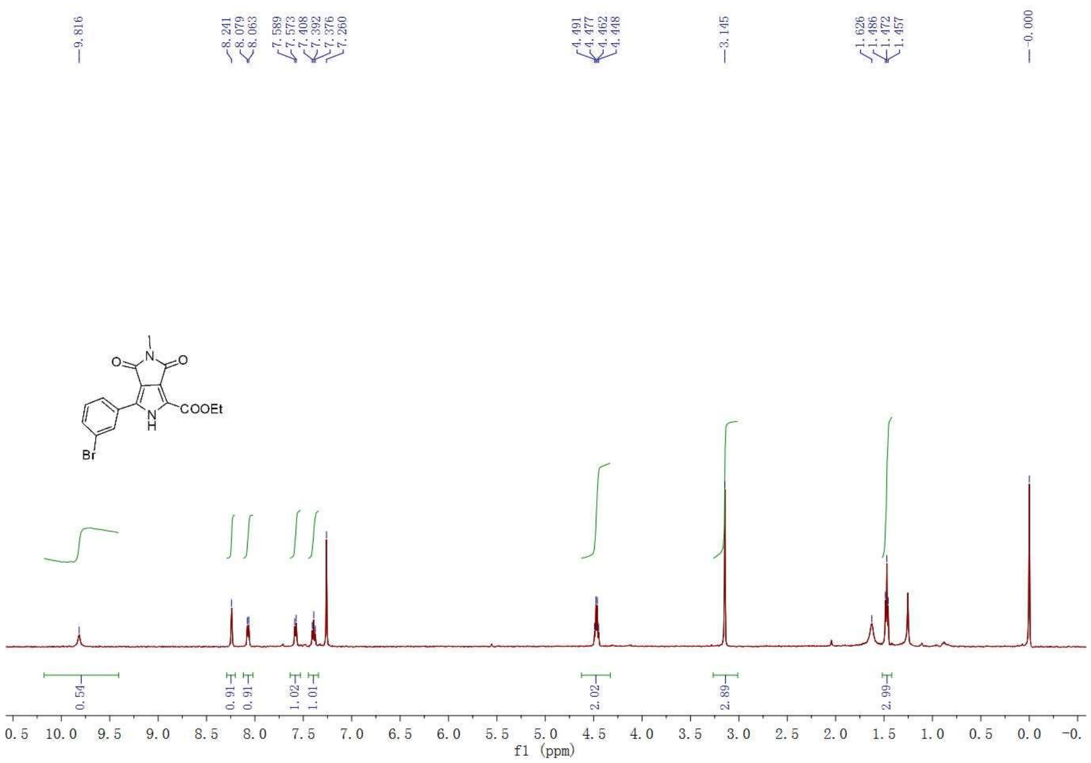

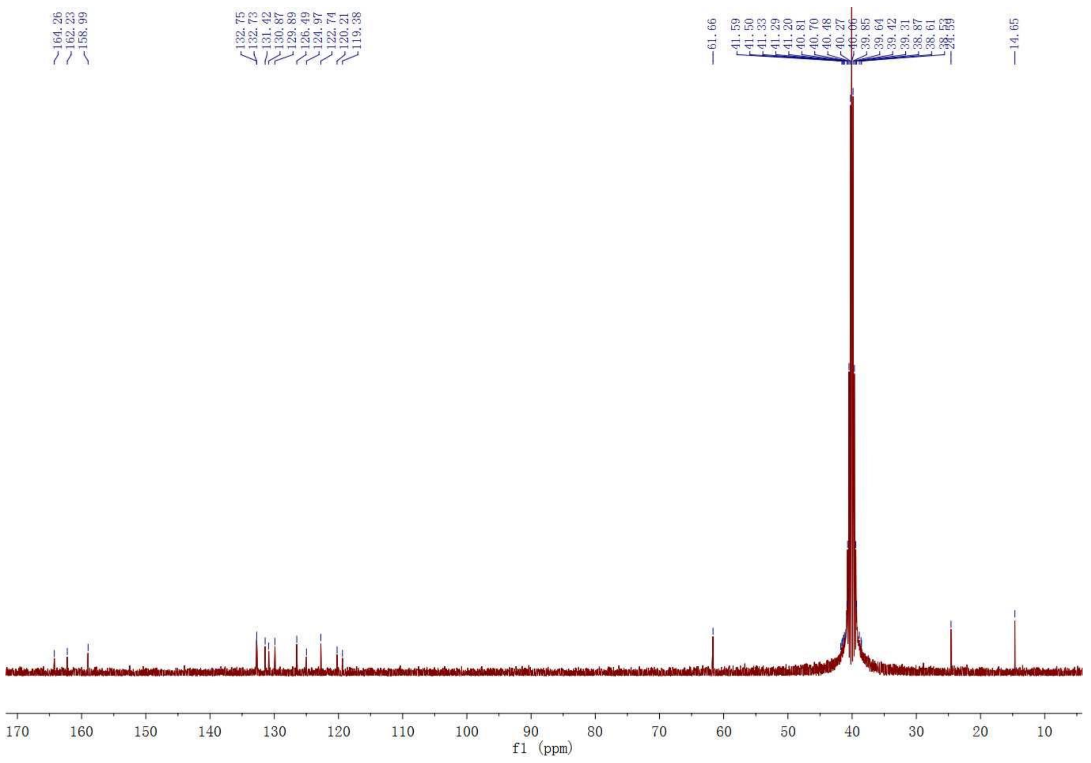

Ethyl 5-methyl-4,6-dioxo-3-(*p*-tolyl)-2,4,5,6-tetrahydropyrrolo[3,4-*c*]pyrrole-1-carboxylate (**12f**)

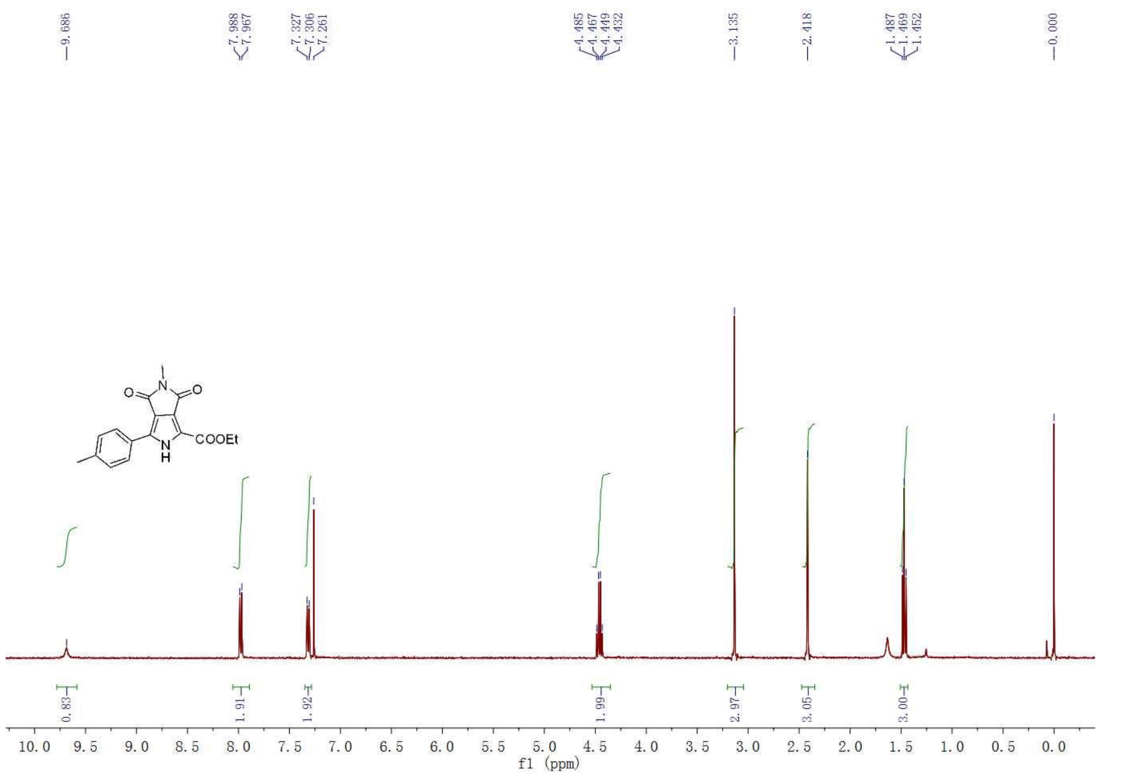

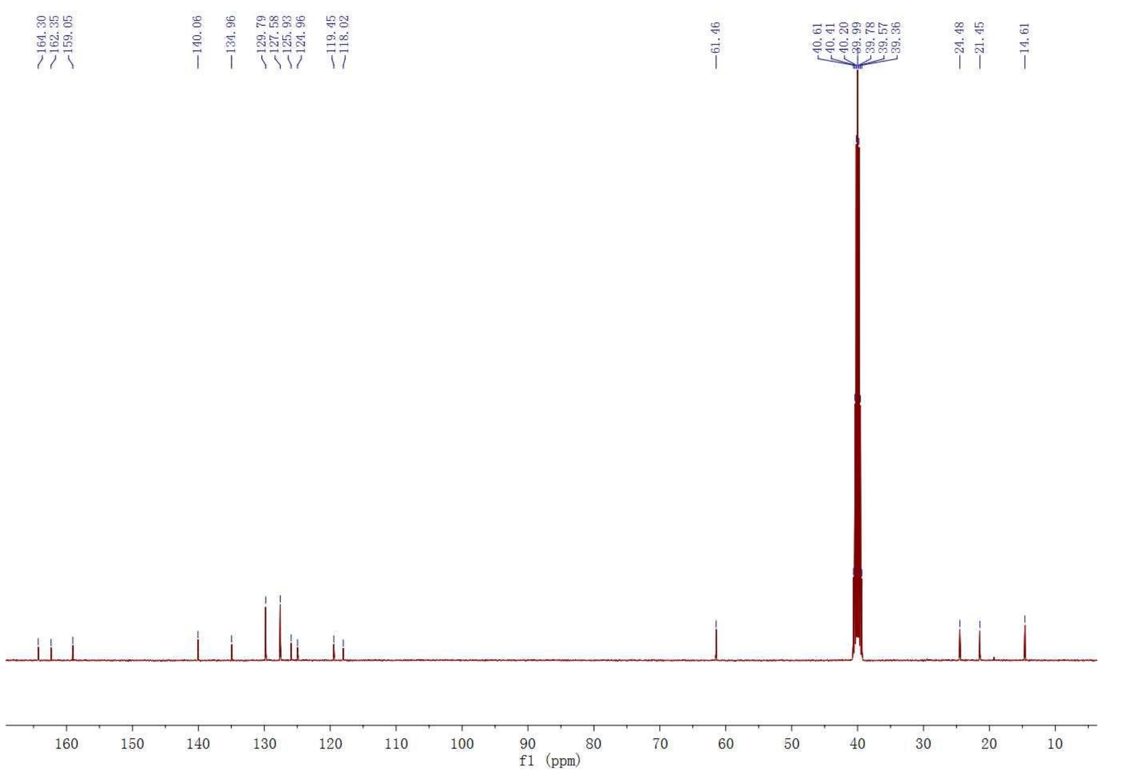

Ethyl 3-(3,4-dimethoxyphenyl)-5-methyl-4,6-dioxo-2,4,5,6-tetrahydropyrrolo[3,4-*c*]pyrrole-1-carboxylate (**12g**)

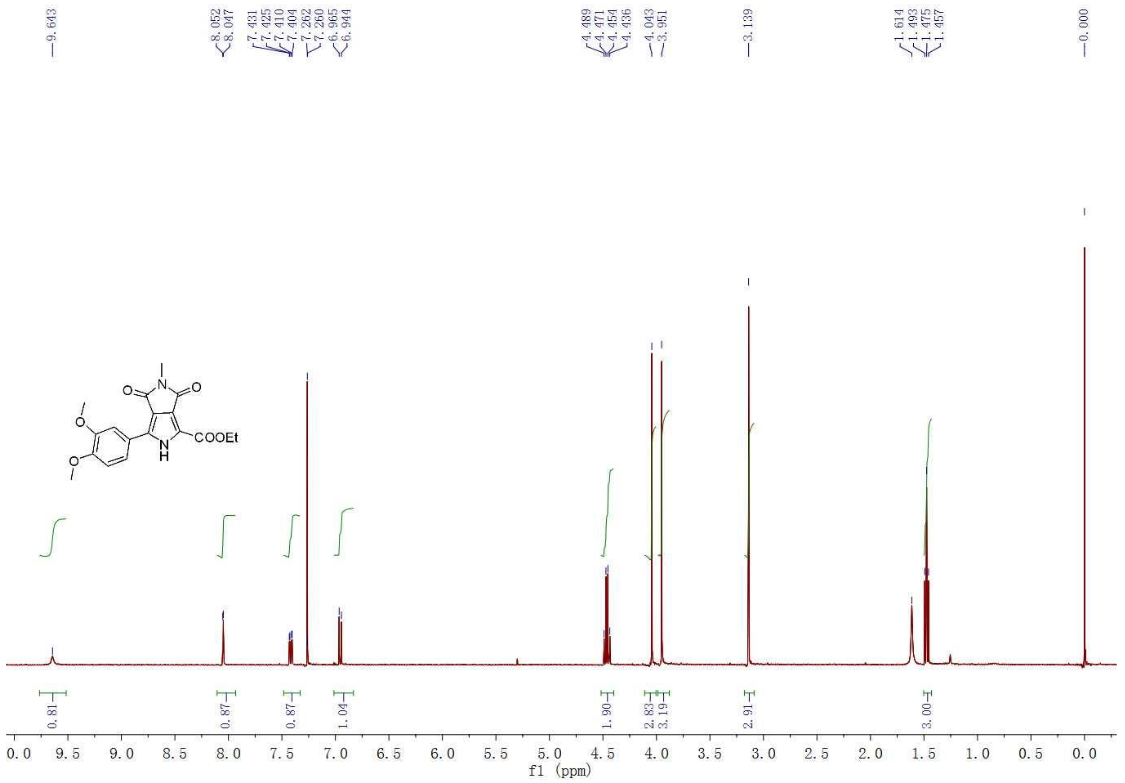

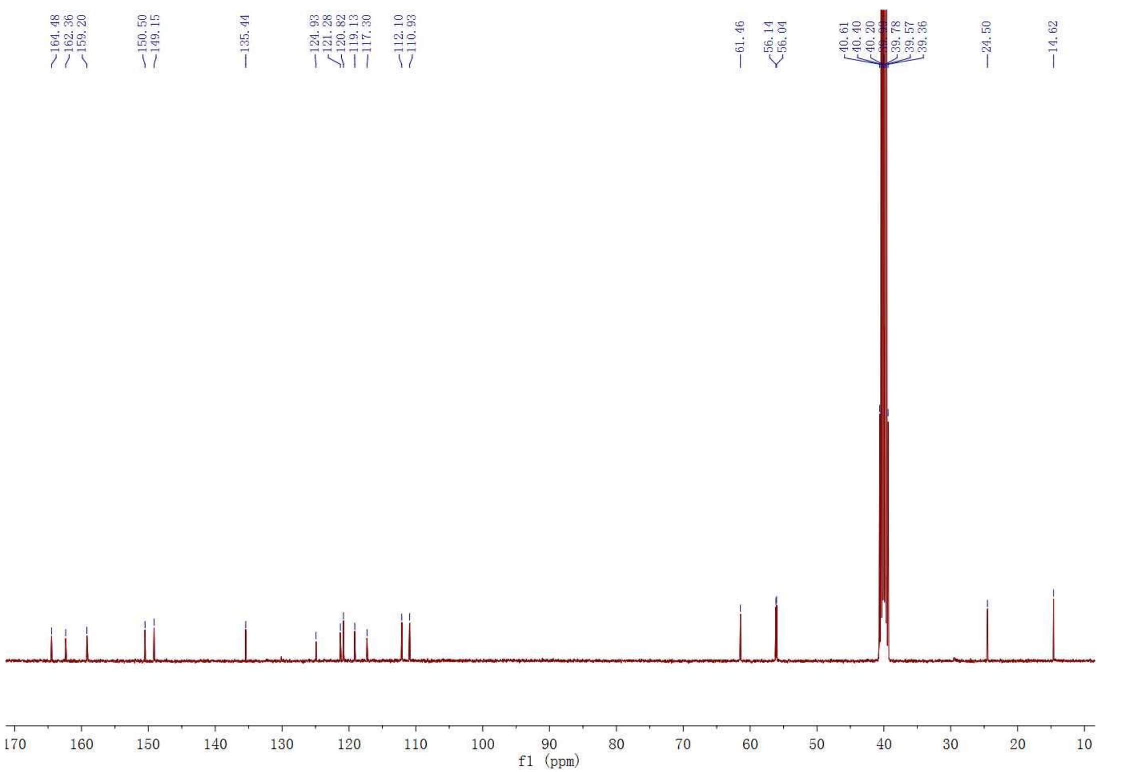

Ethyl (*E*)-5-methyl-4,6-dioxo-3-styryl-2,4,5,6-tetrahydropyrrolo[3,4-*c*]pyrrole-1-carboxylate (**12h**)

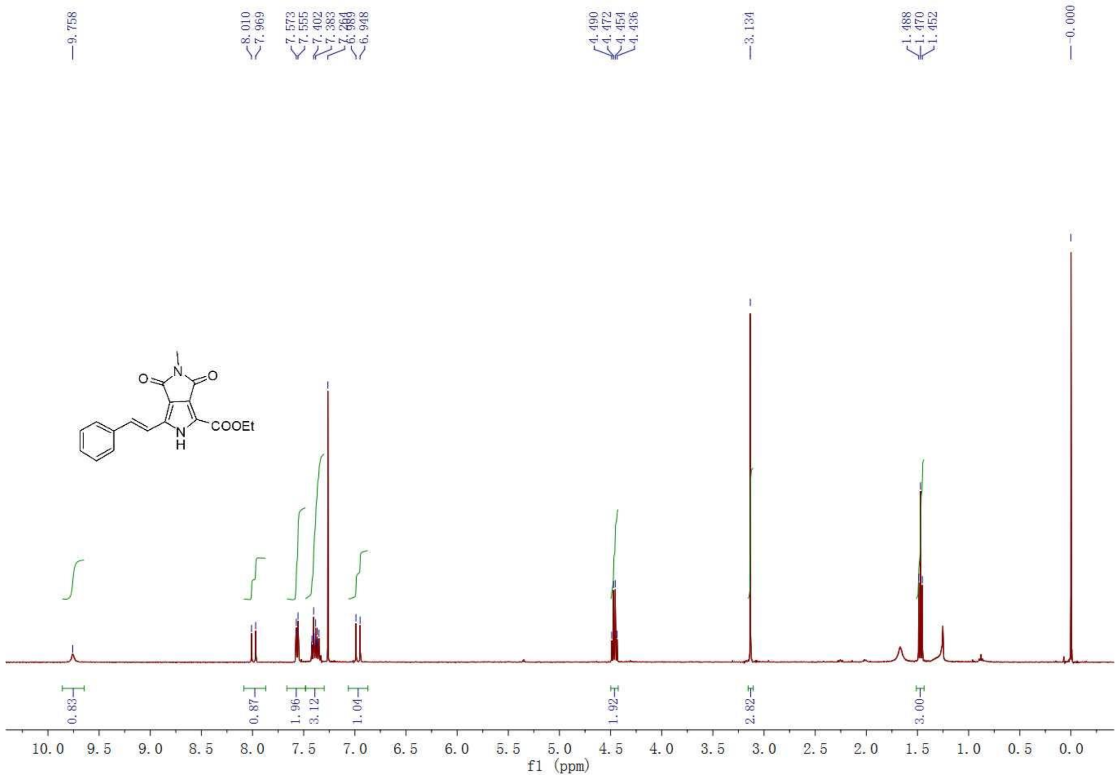

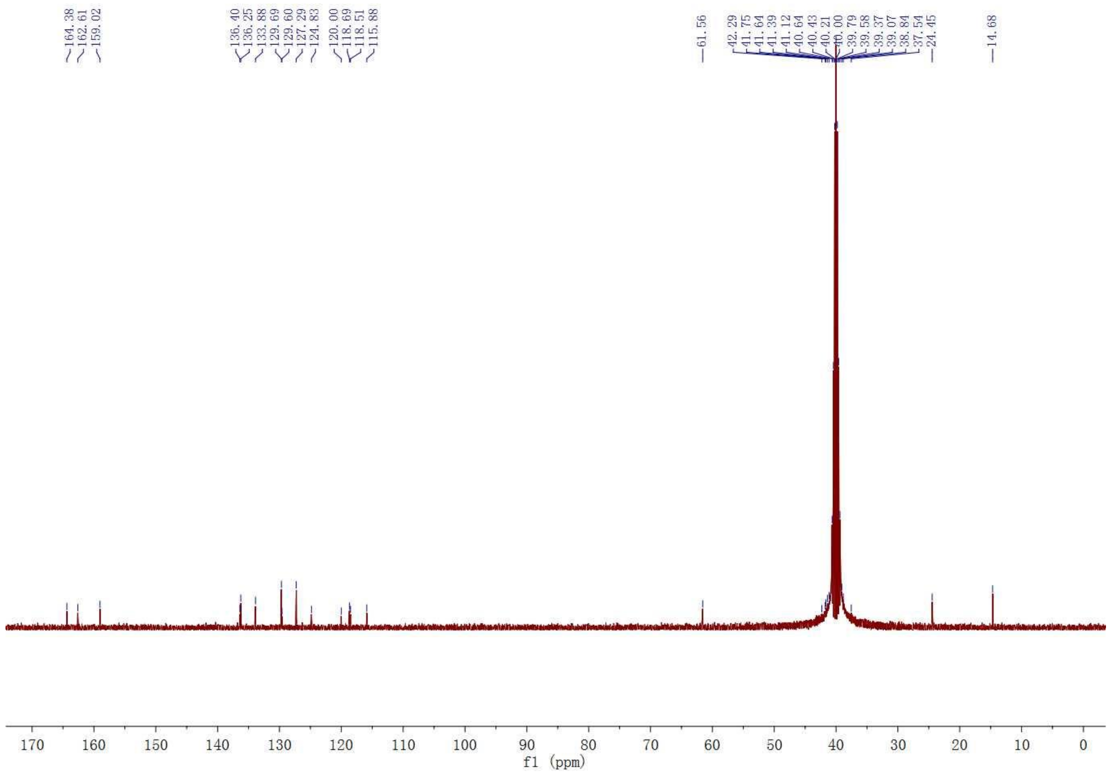

Ethyl 3-(furan-2-yl)-5-methyl-4,6-dioxo-2,4,5,6-tetrahydropyrrolo[3,4-*c*]pyrrole-1-carboxylate (**12i**)

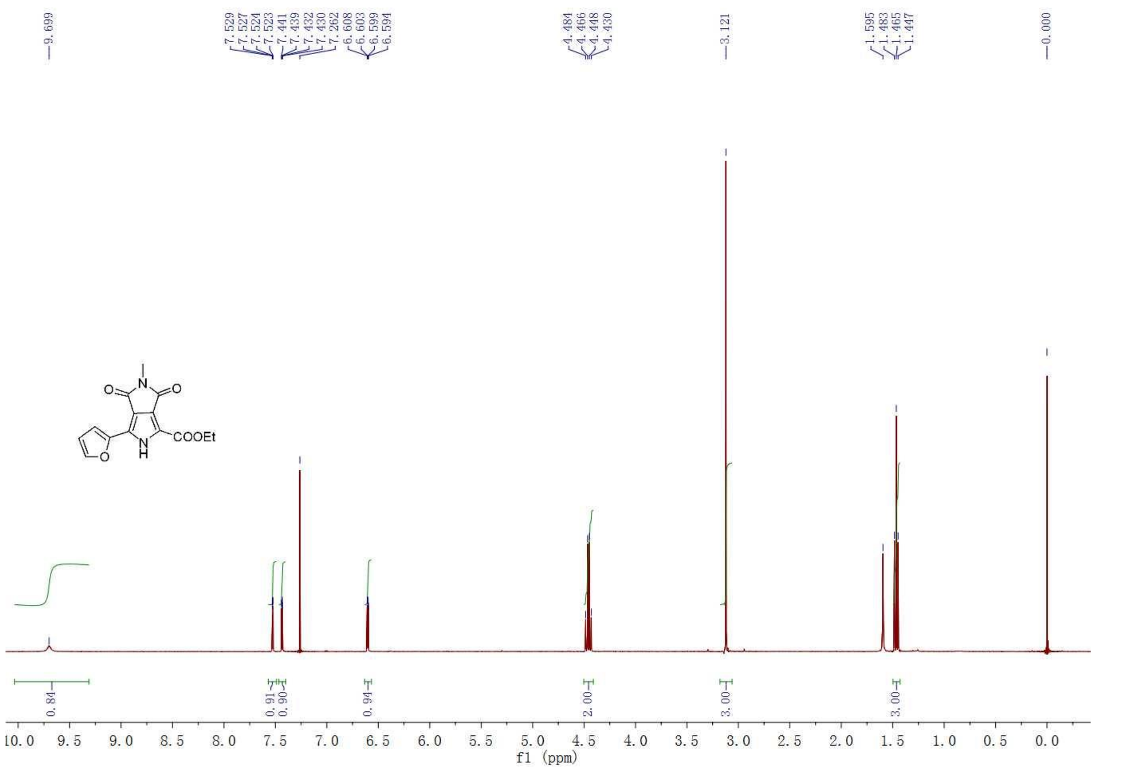

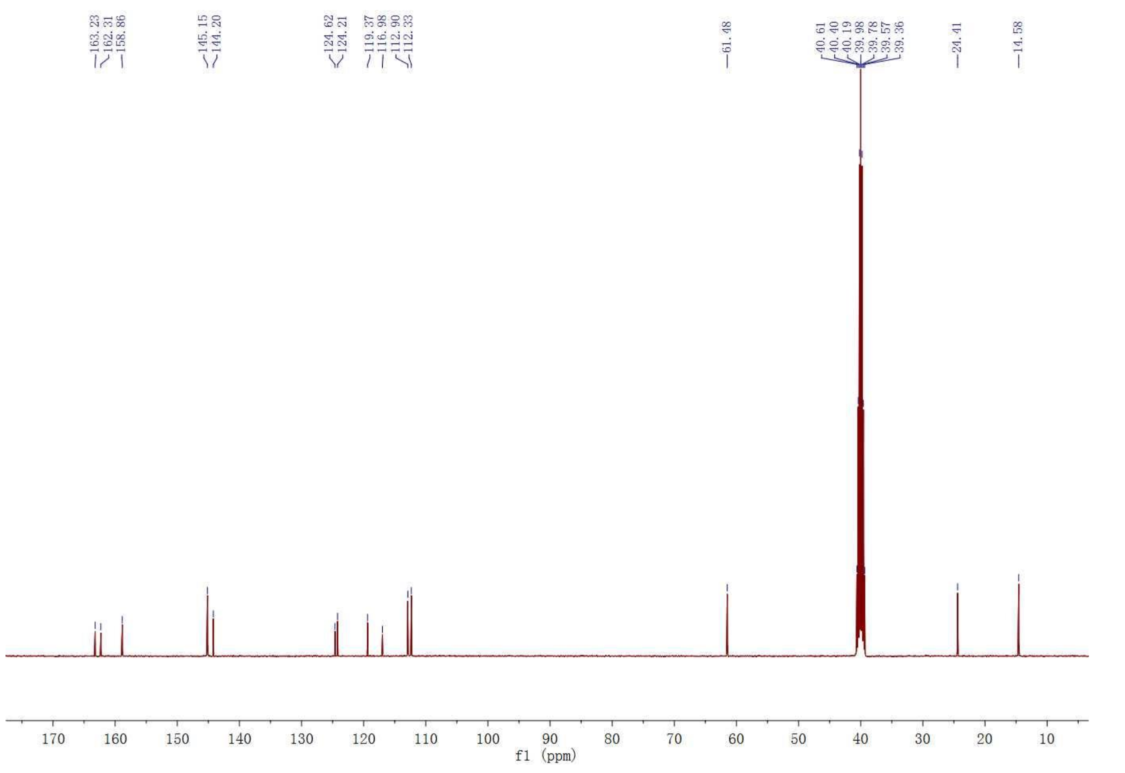

Ethyl 5-methyl-4,6-dioxo-3-(thiophen-2-yl)-2,4,5,6-tetrahydropyrrolo[3,4-*c*]pyrrole-1-carboxylate (**12j**)

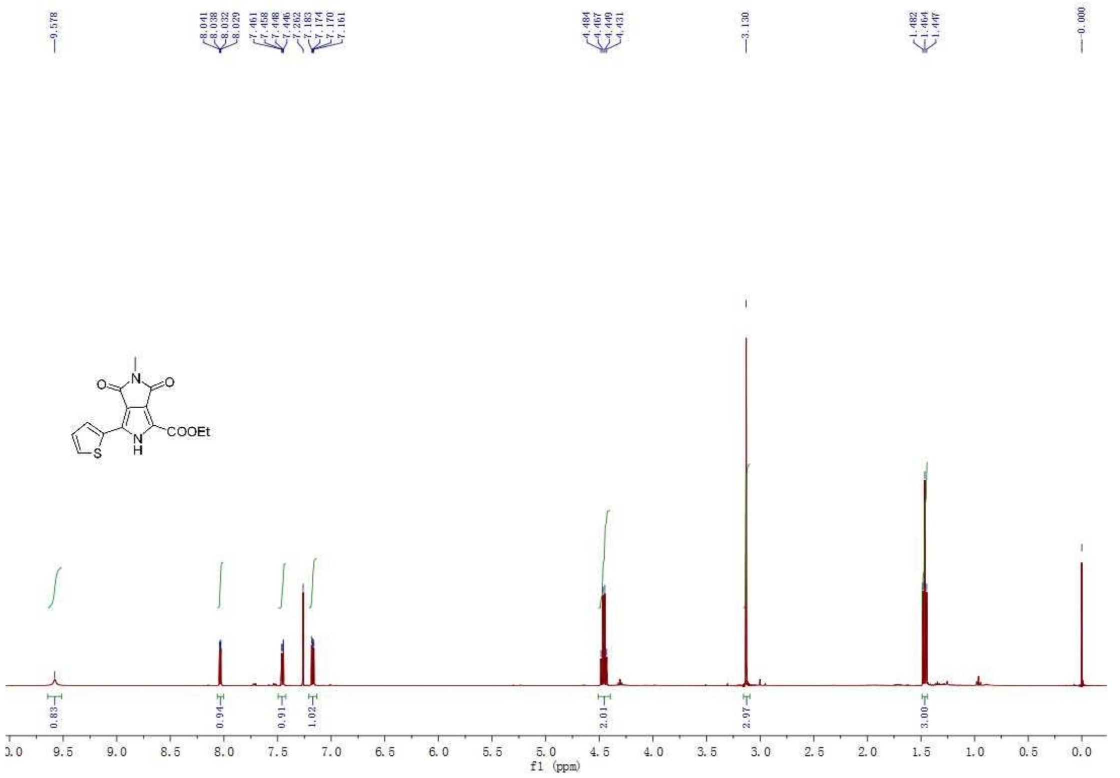

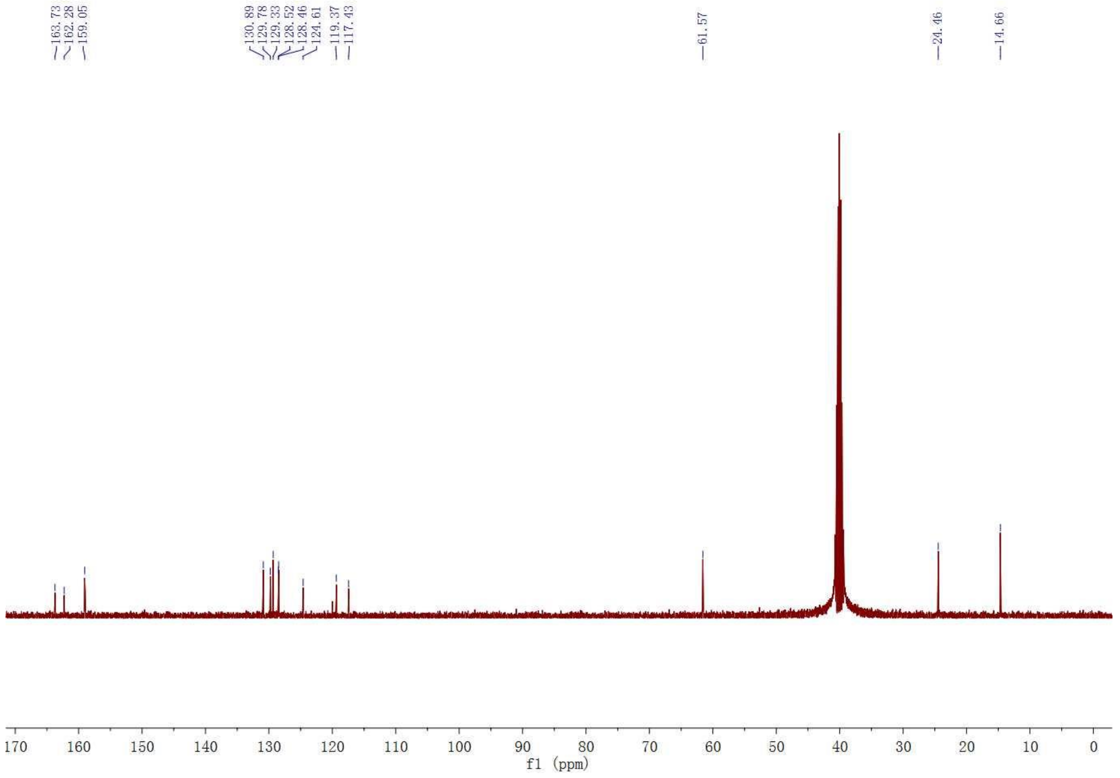

Ethyl 5-ethyl-4,6-dioxo-3-phenyl-2,4,5,6-tetrahydropyrrolo[3,4-*c*]pyrrole-1-carboxylate (**12k**)

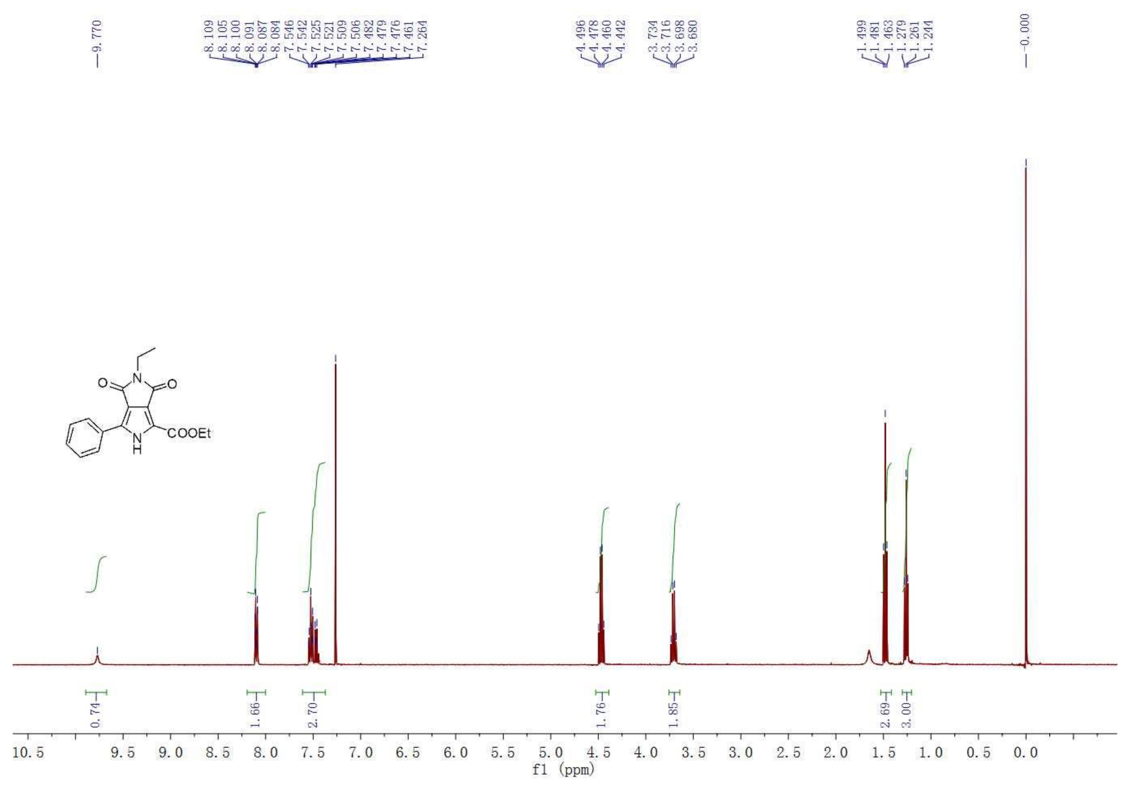

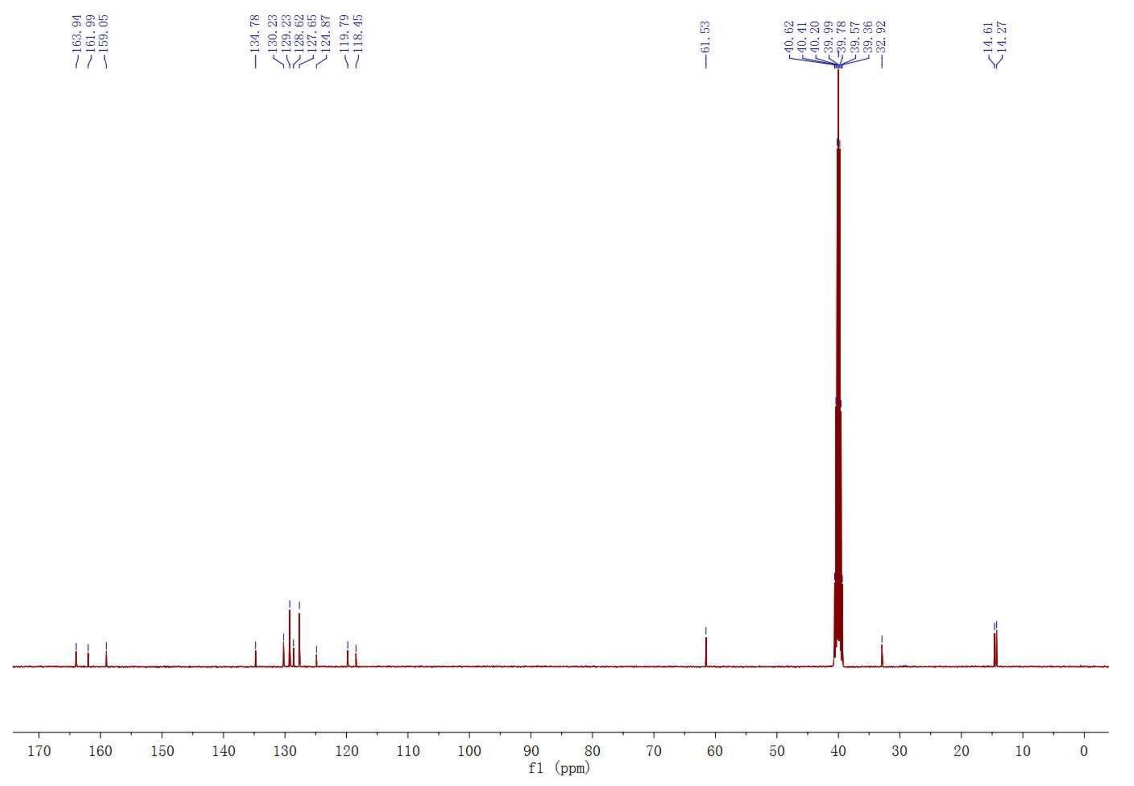

N^2,N^3,N^4-Trimethyl-5-phenyl-1*H*-pyrrole-2,3,4-tricarboxamide (**13a**)

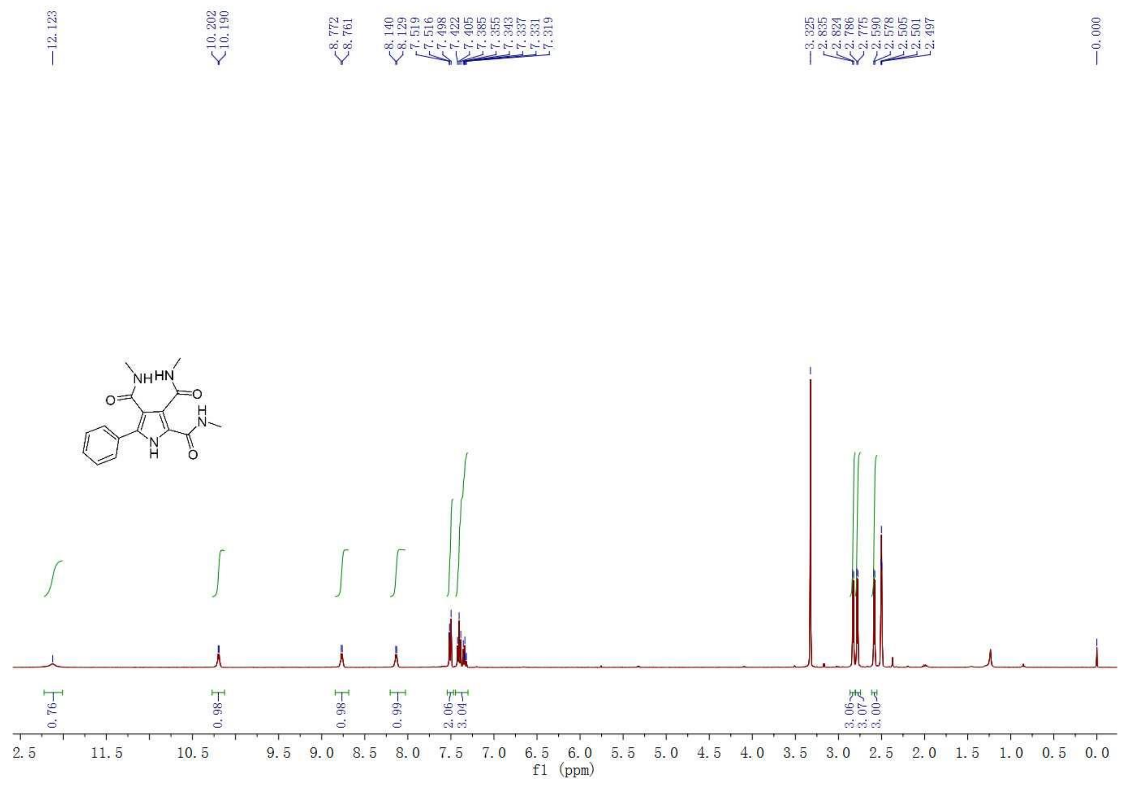

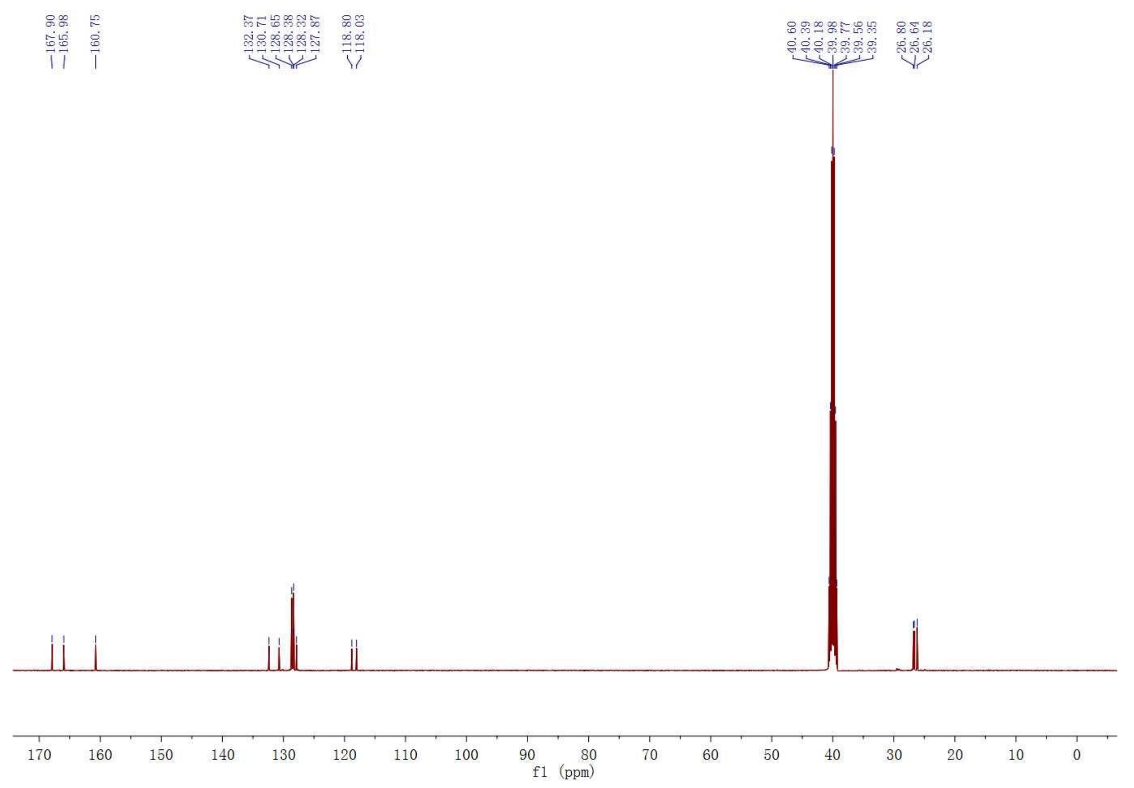

5-(2-Chlorophenyl)-N^2,N^3,N^4-trimethyl-1*H*-pyrrole-2,3,4-tricarboxamide (**13b**)

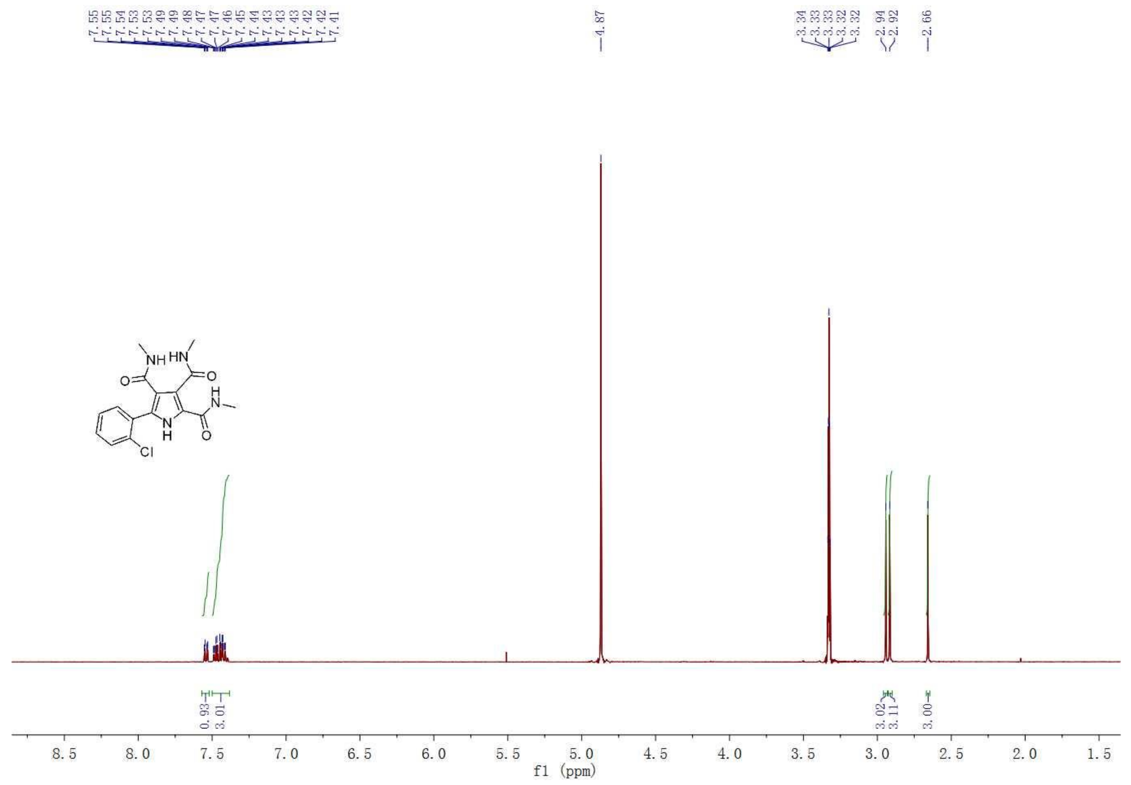

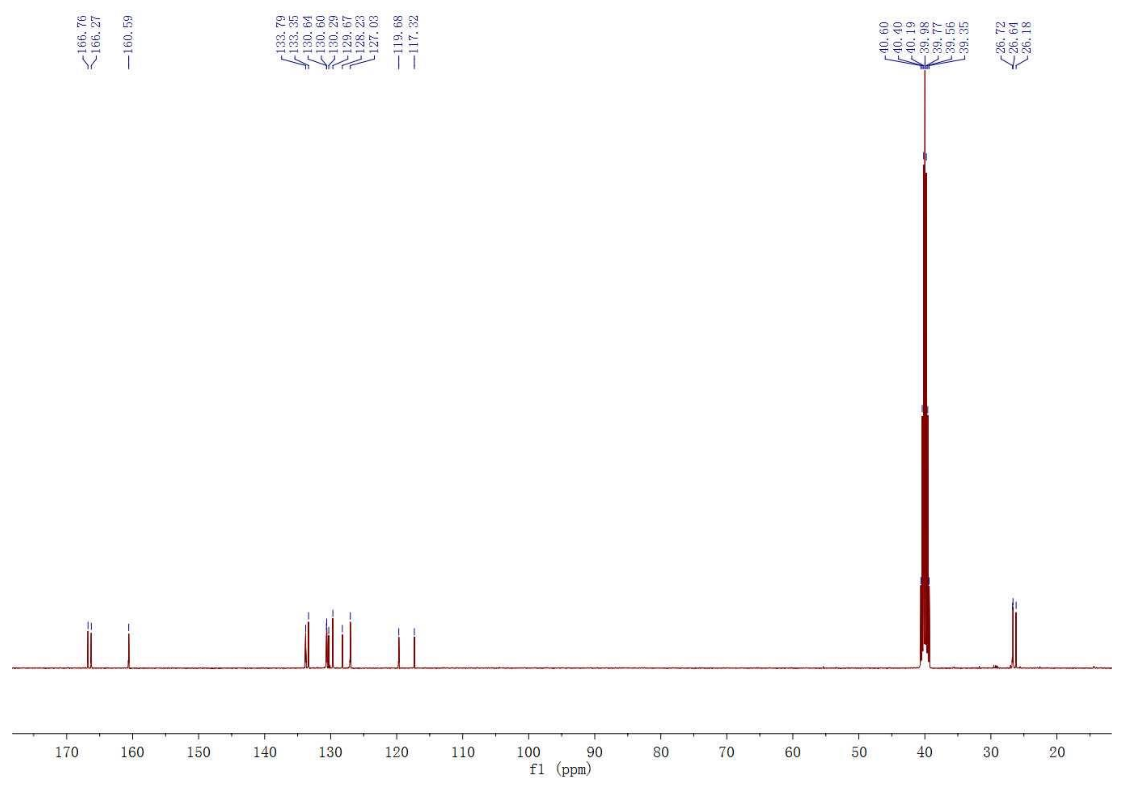

5-(3-Chlorophenyl)-N^2,N^3,N^4-trimethyl-1H-pyrrole-2,3,4-tricarboxamide (**13c**)

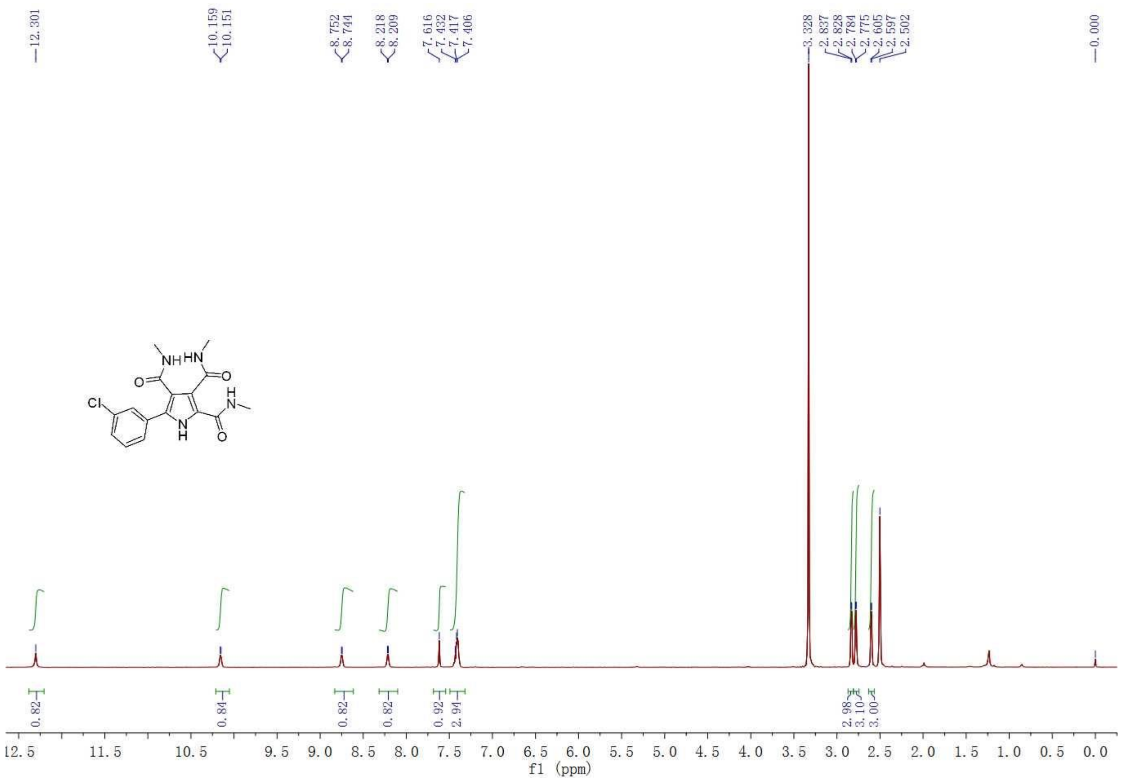

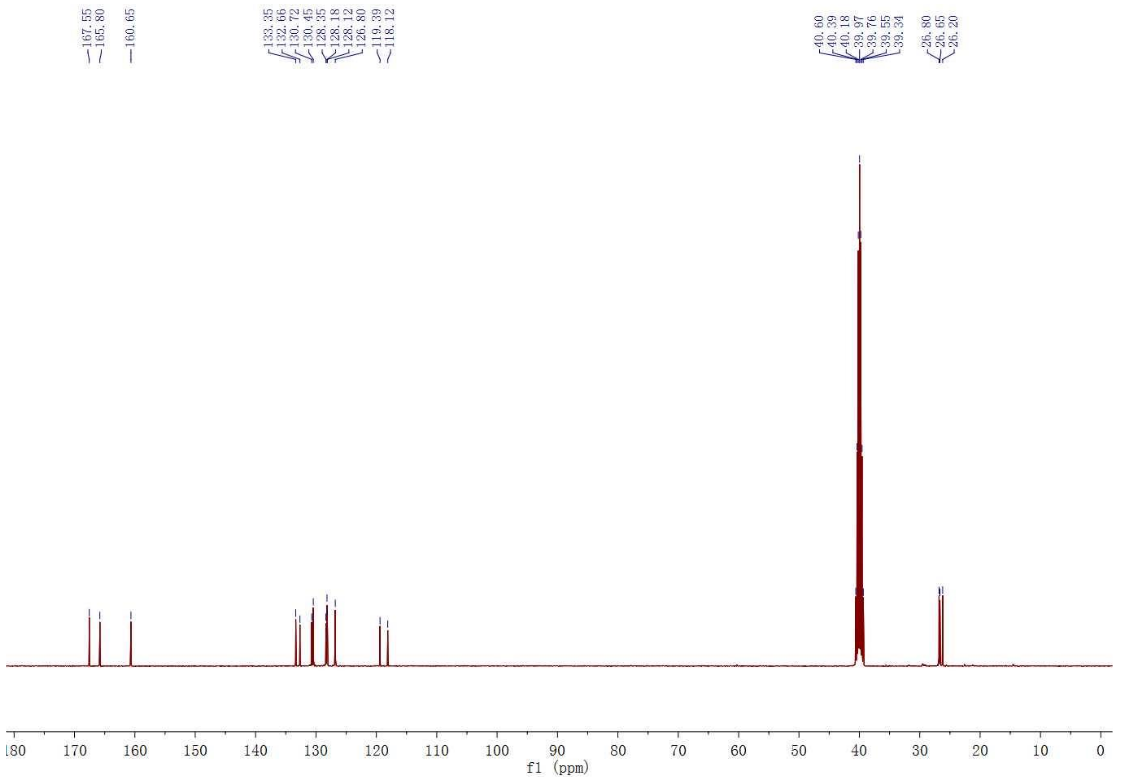

5-(4-Chlorophenyl)-N^2,N^3,N^4-trimethyl-1*H*-pyrrole-2,3,4-tricarboxamide (**13d**)

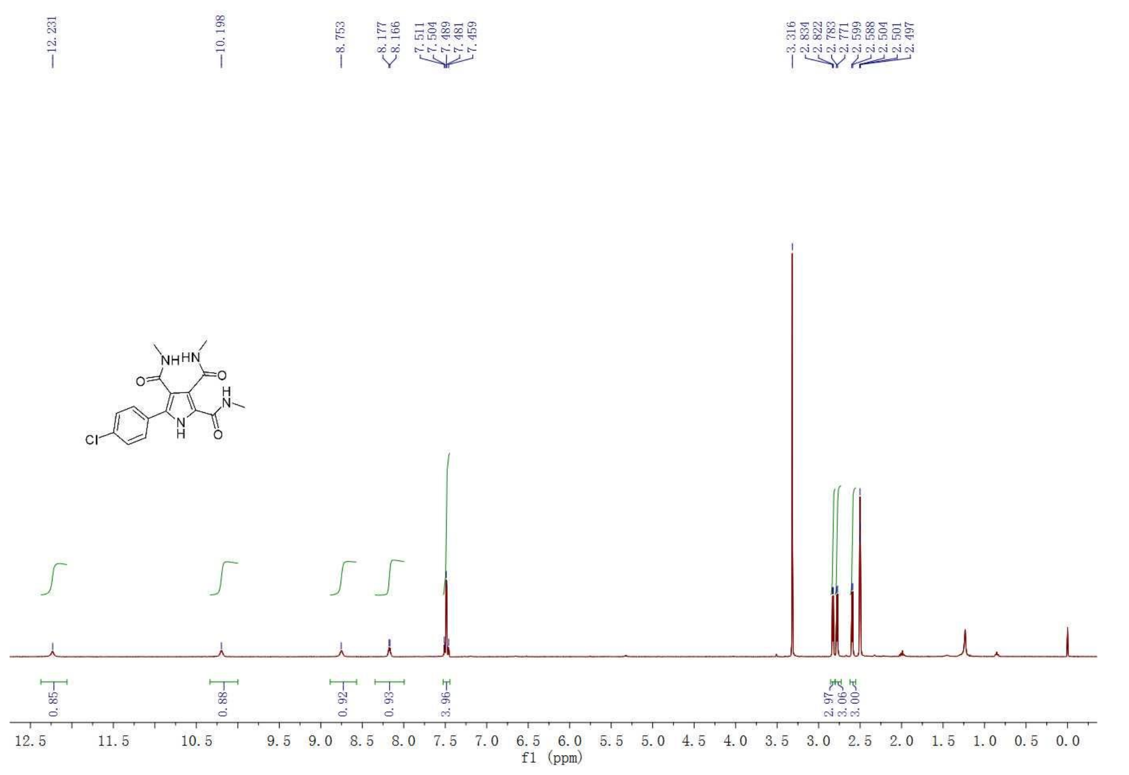

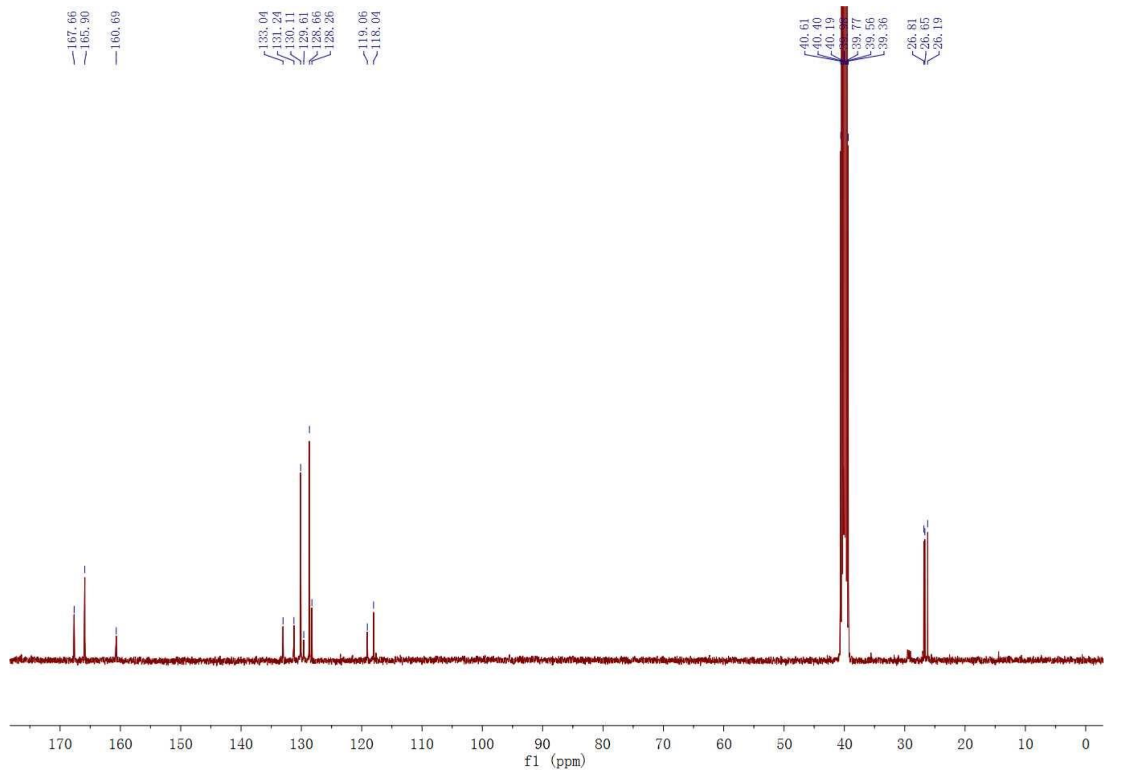

5-(3-Bromophenyl)-N^2,N^3,N^4-trimethyl-1H-pyrrole-2,3,4-tricarboxamide (**13e**)

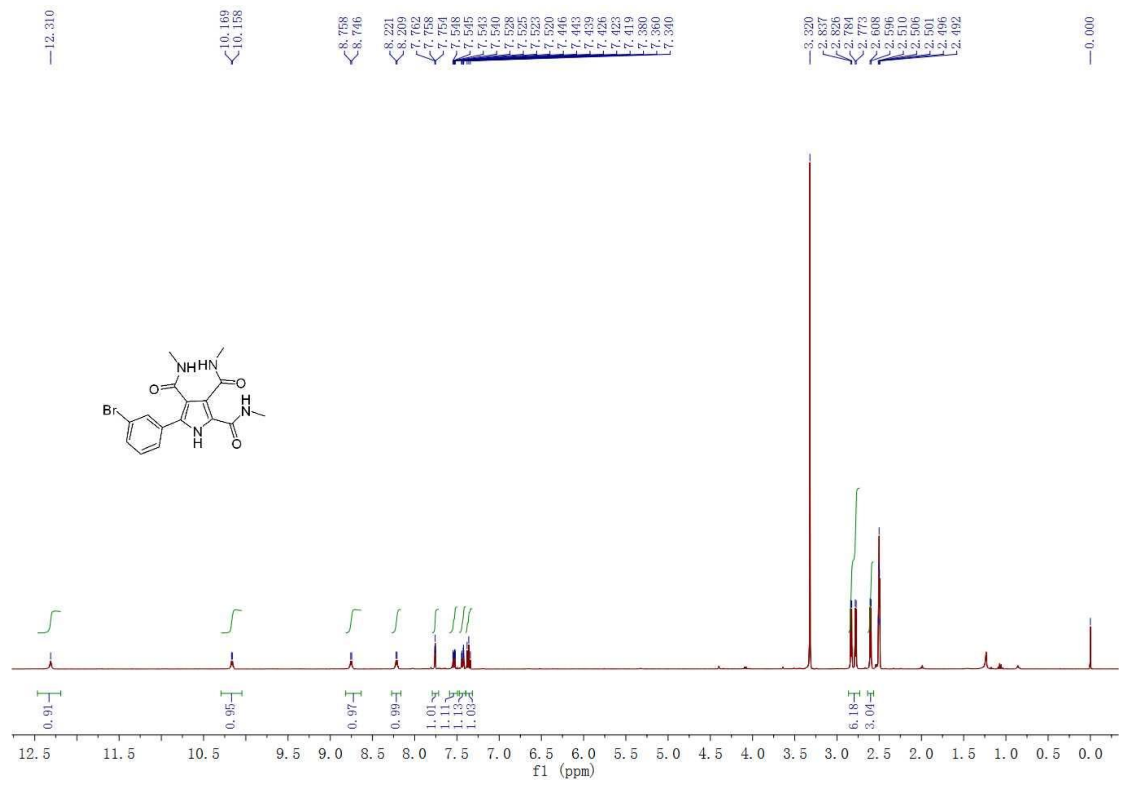

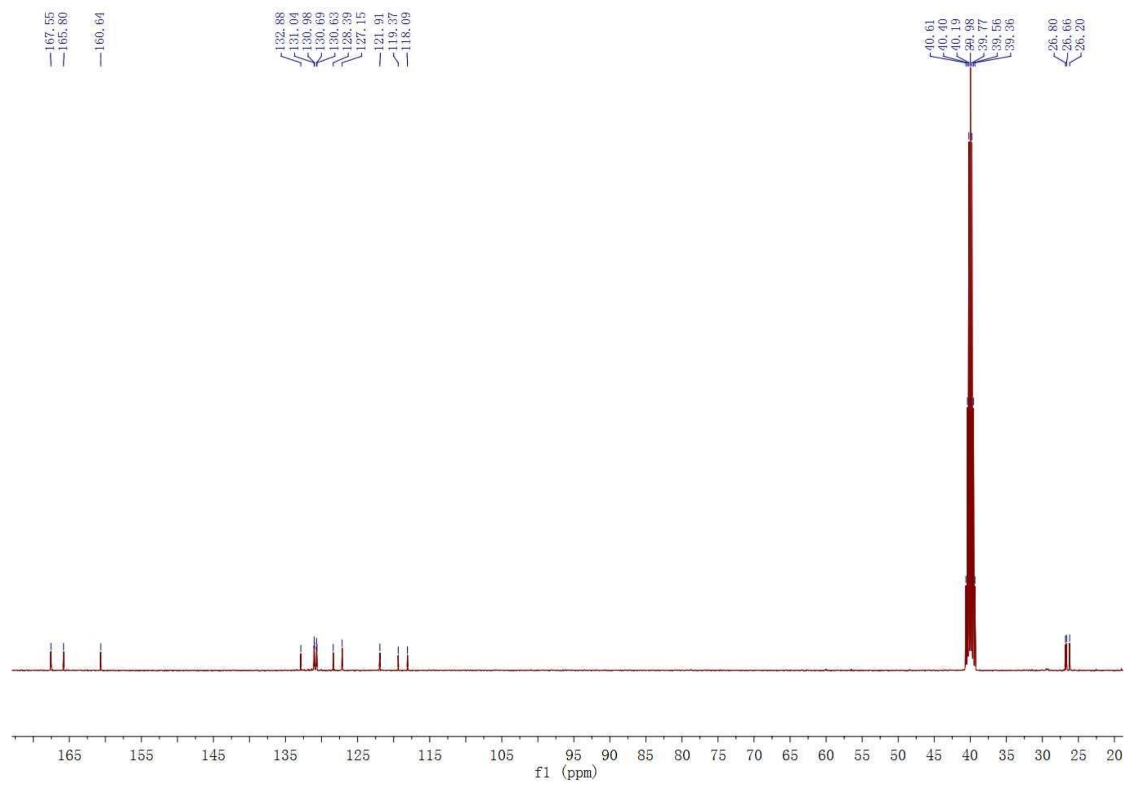

N^2,N^3,N^4-Trimethyl-5-(*p*-tolyl)-1*H*-pyrrole-2,3,4-tricarboxamide (**13f**)

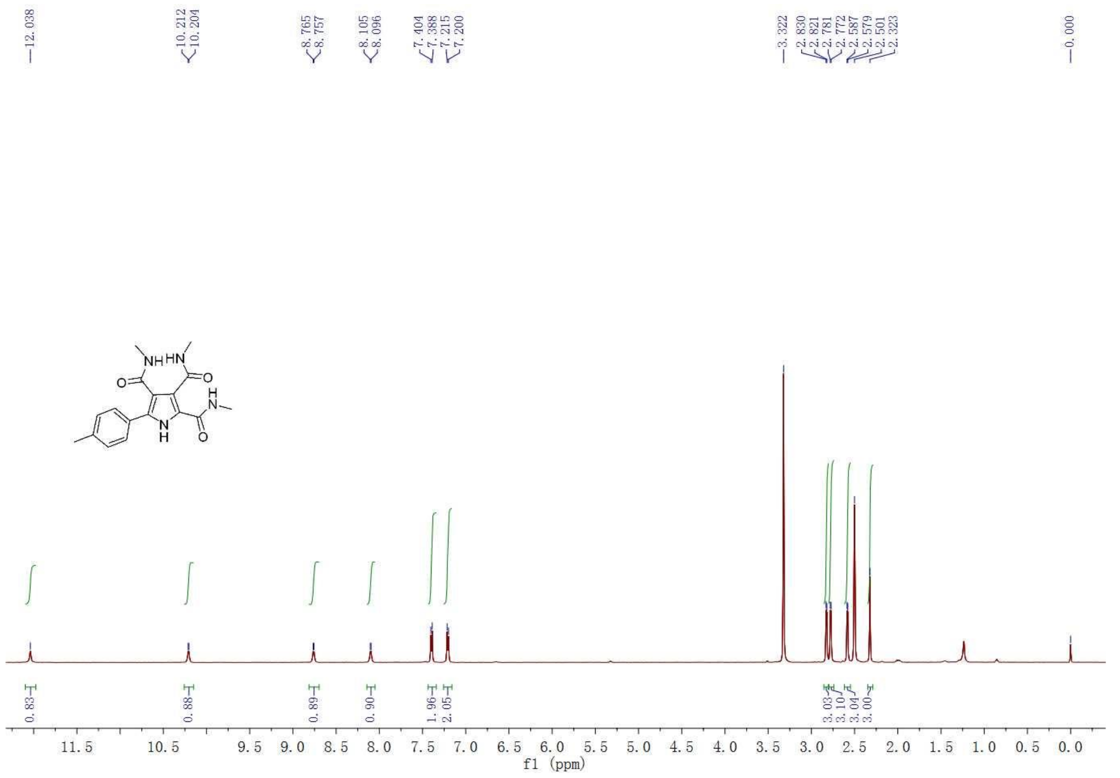

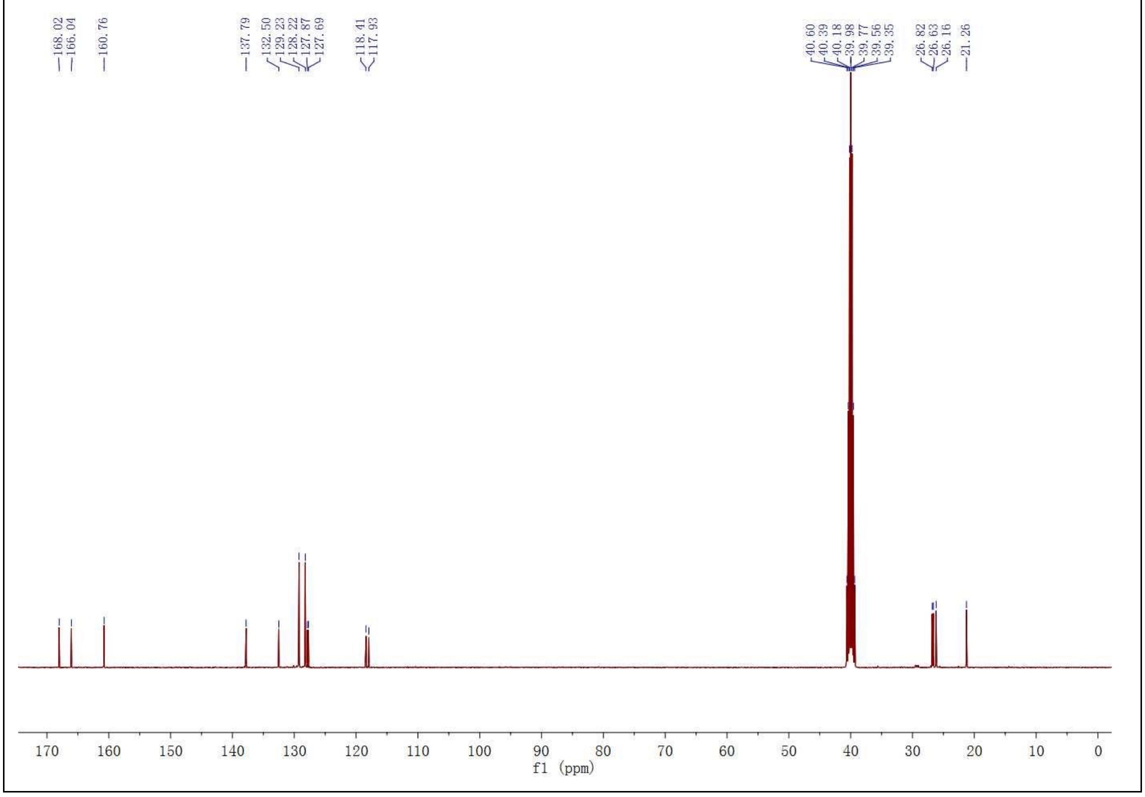

5-(3,4-Dimethoxyphenyl)-N^2,N^3,N^4-trimethyl-1*H*-pyrrole-2,3,4-tricarboxamide (**13g**)

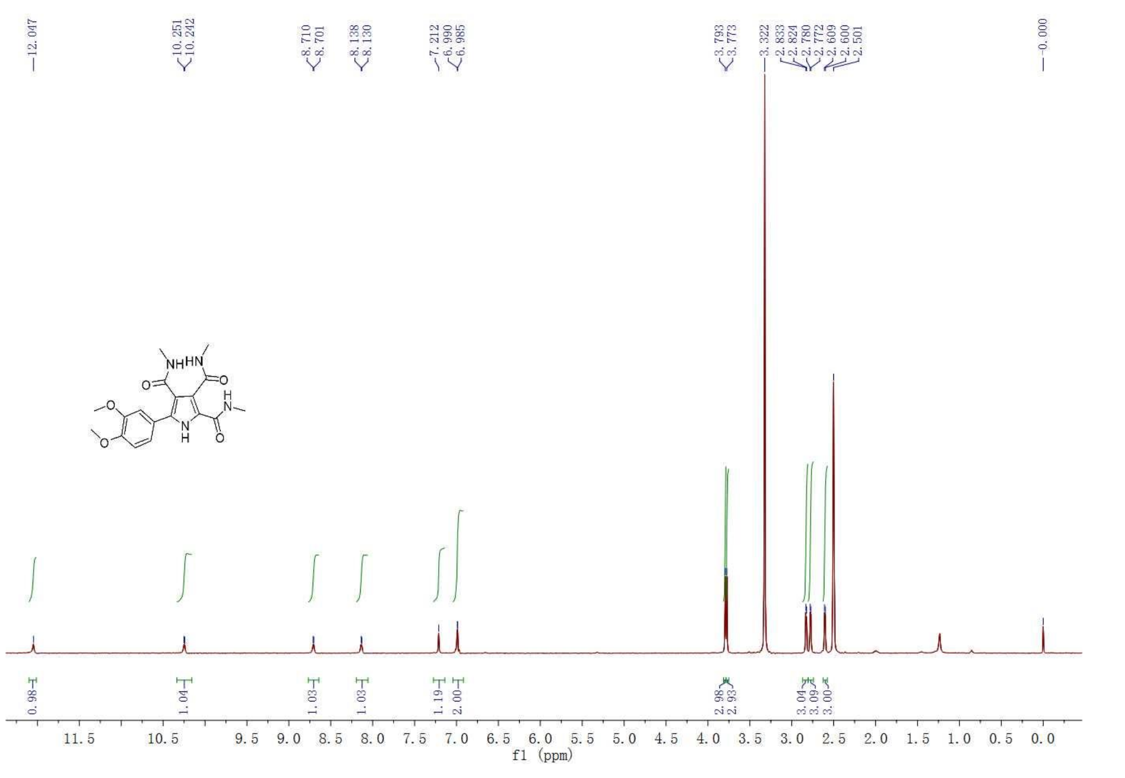

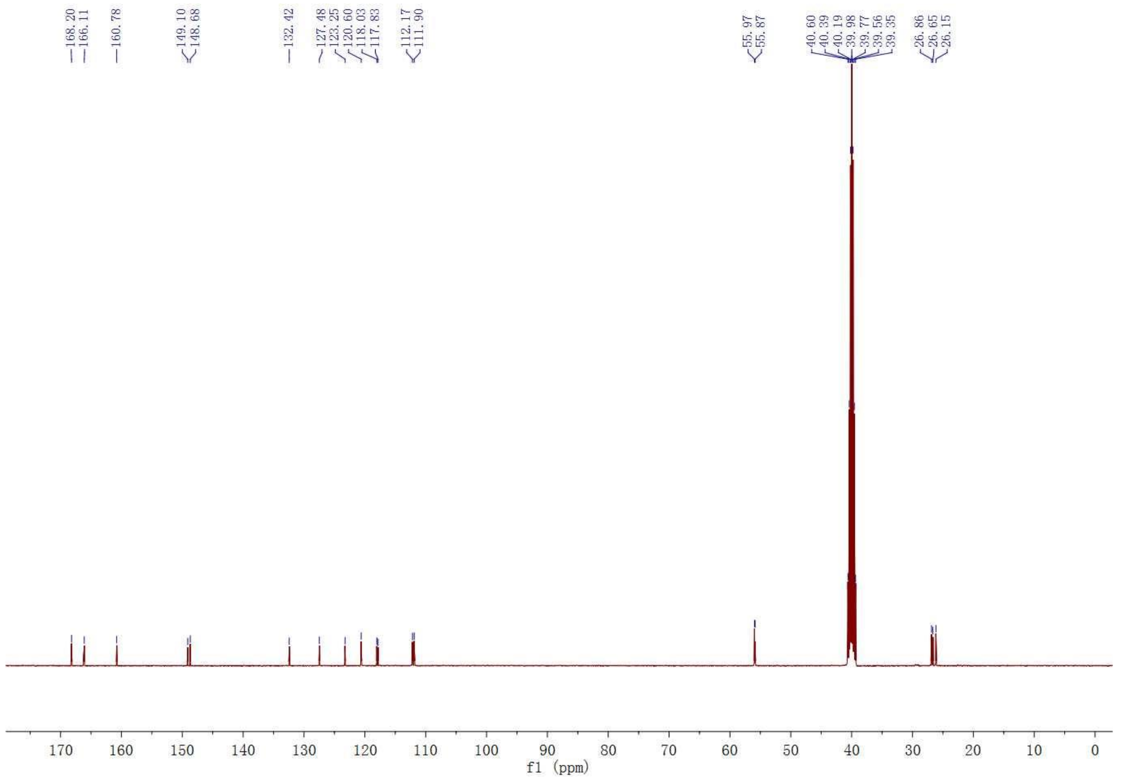

(*E*)-N^2,N^3,N^4-Trimethyl-5-styryl-1*H*-pyrrole-2,3,4-tricarboxamide (**13h**)

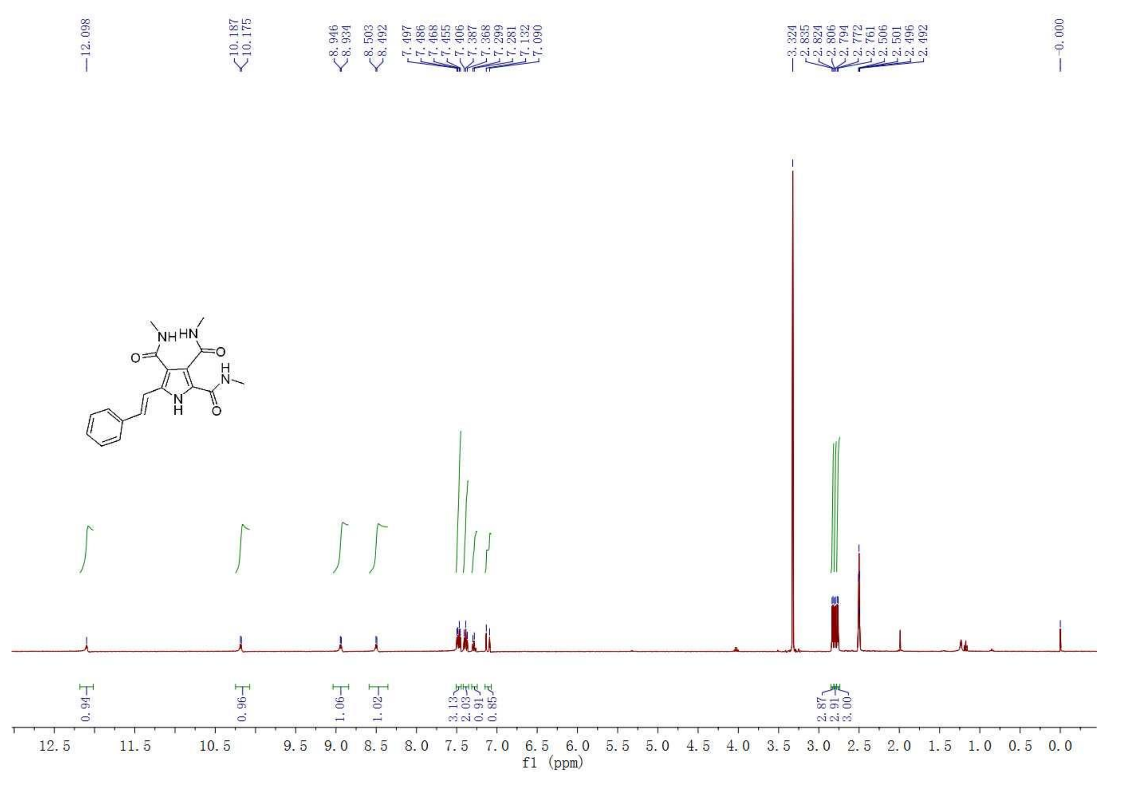

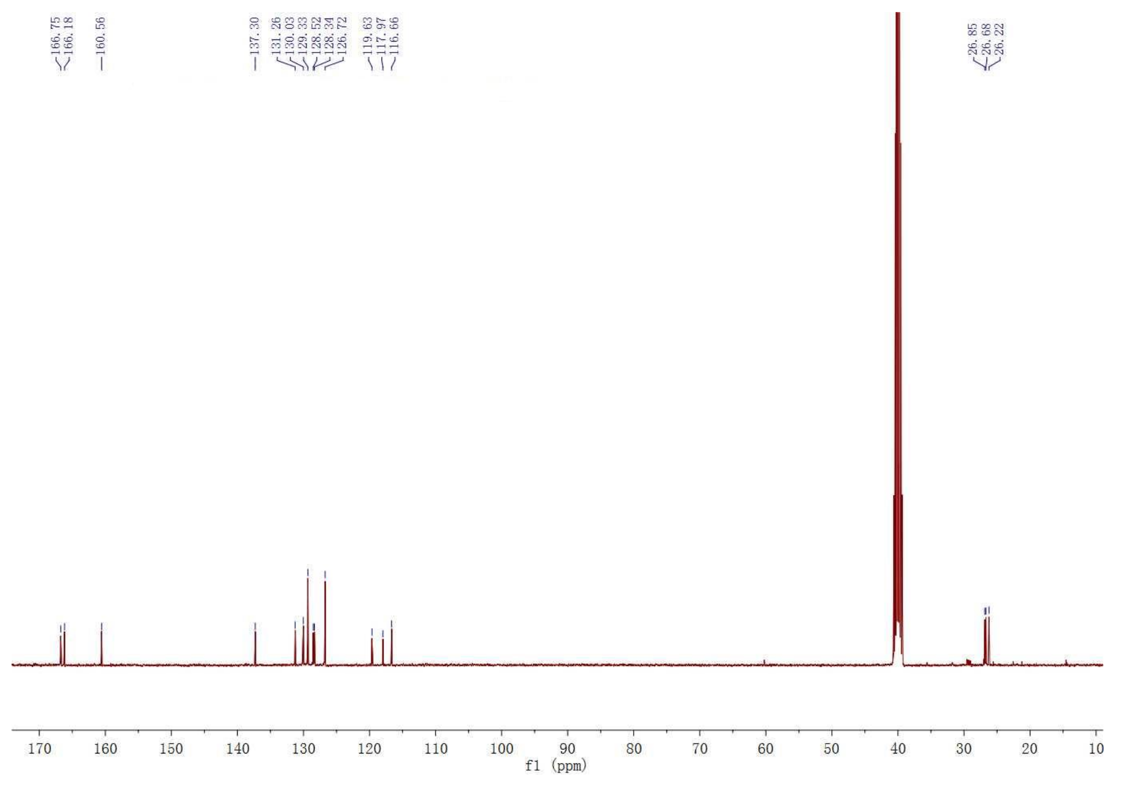

5-(Furan-2-yl)-N^2,N^3,N^4-trimethyl-1*H*-pyrrole-2,3,4-tricarboxamide (**13i**)

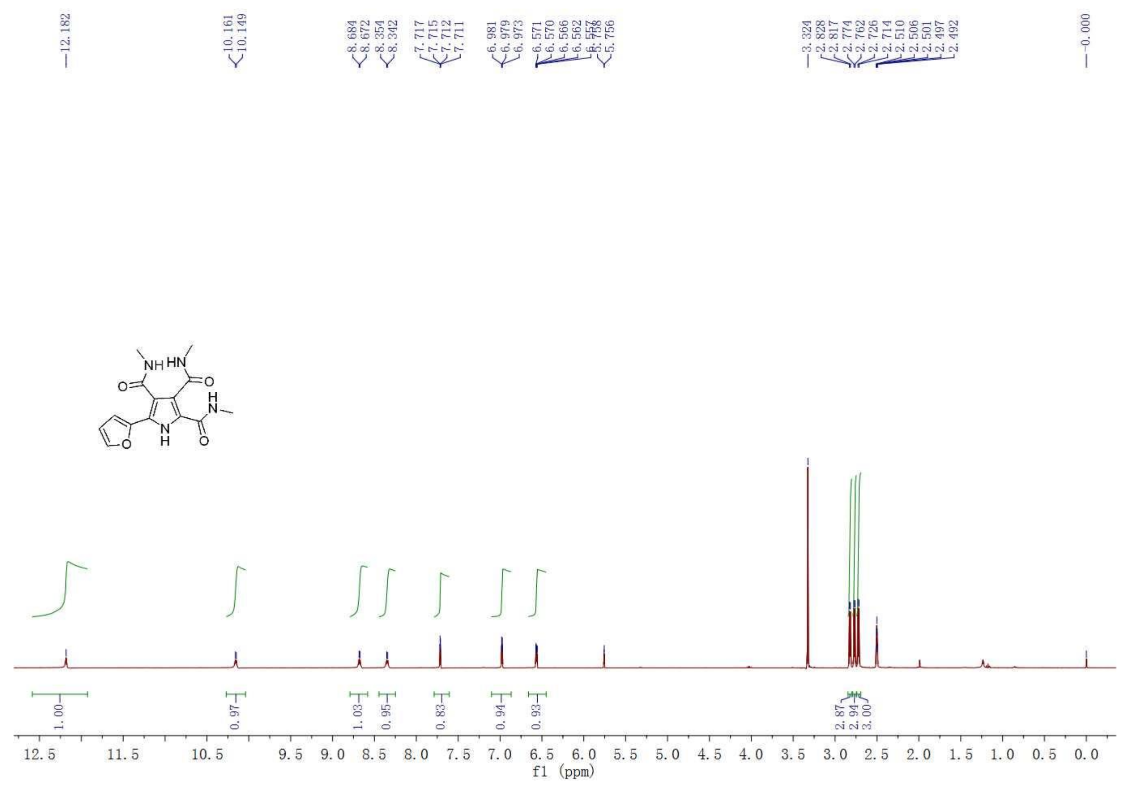

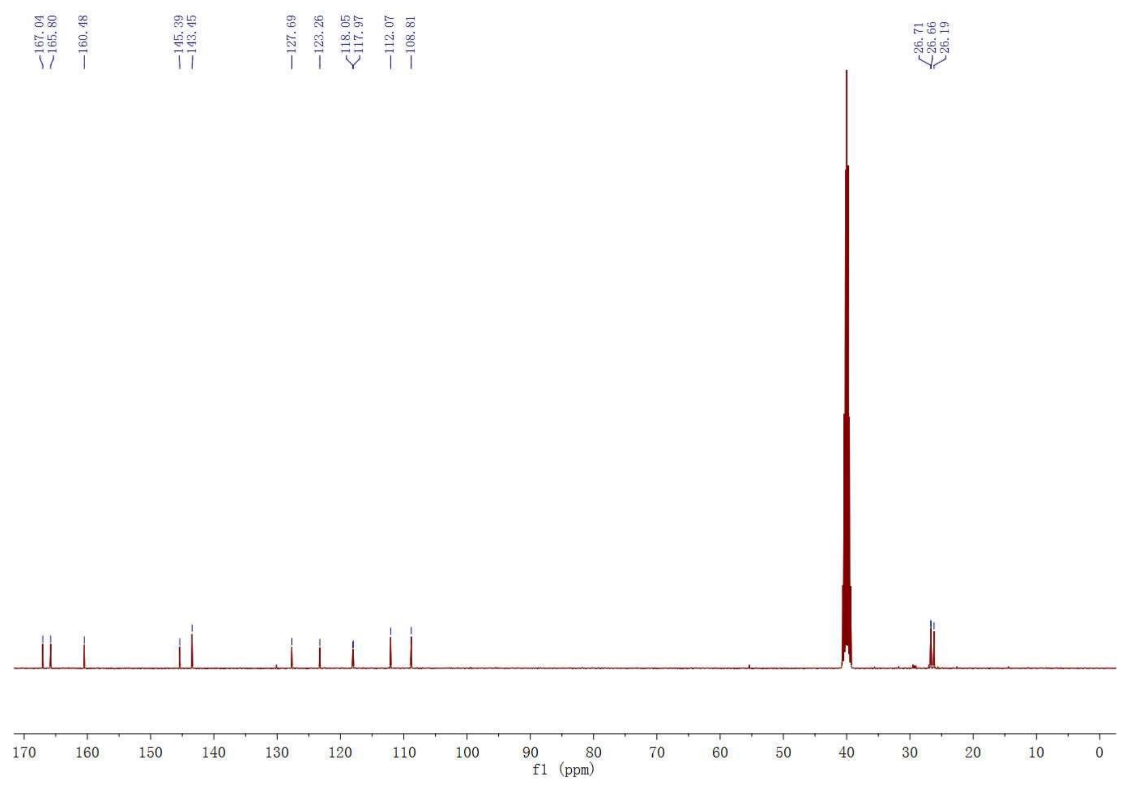

N^2,N^3,N^4-Trimethyl-5-(thiophen-2-yl)-1*H*-pyrrole-2,3,4-tricarboxamide (**13j**)

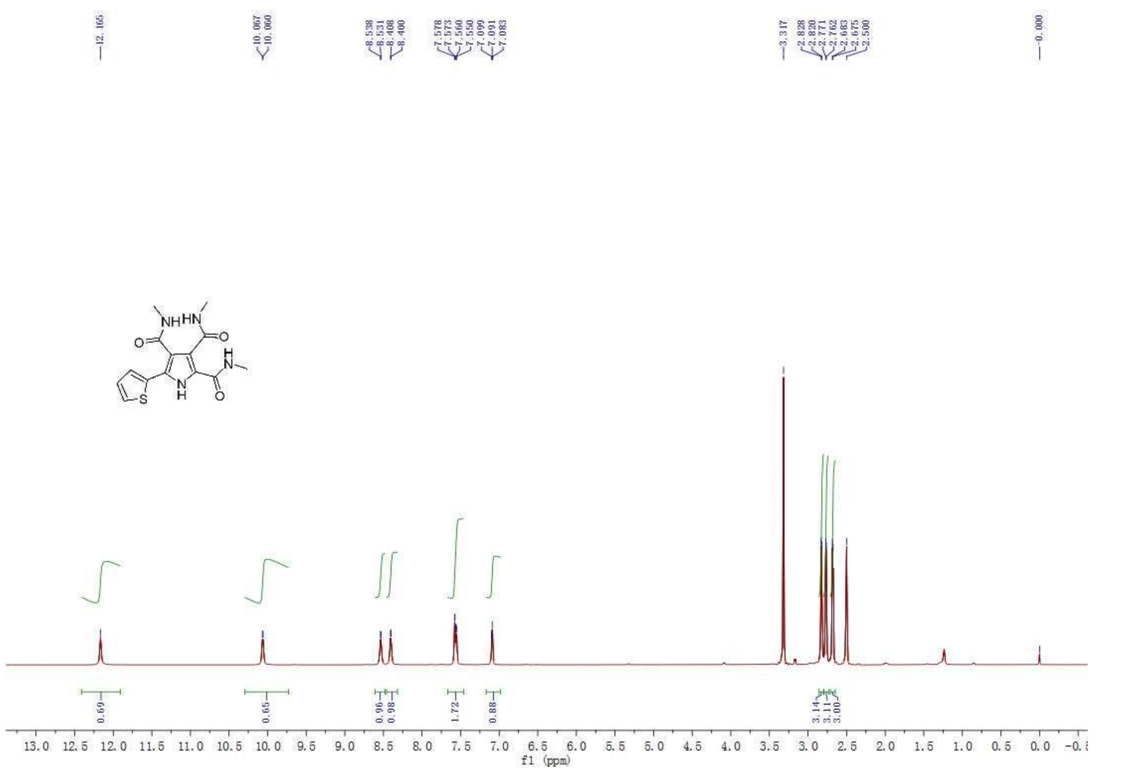

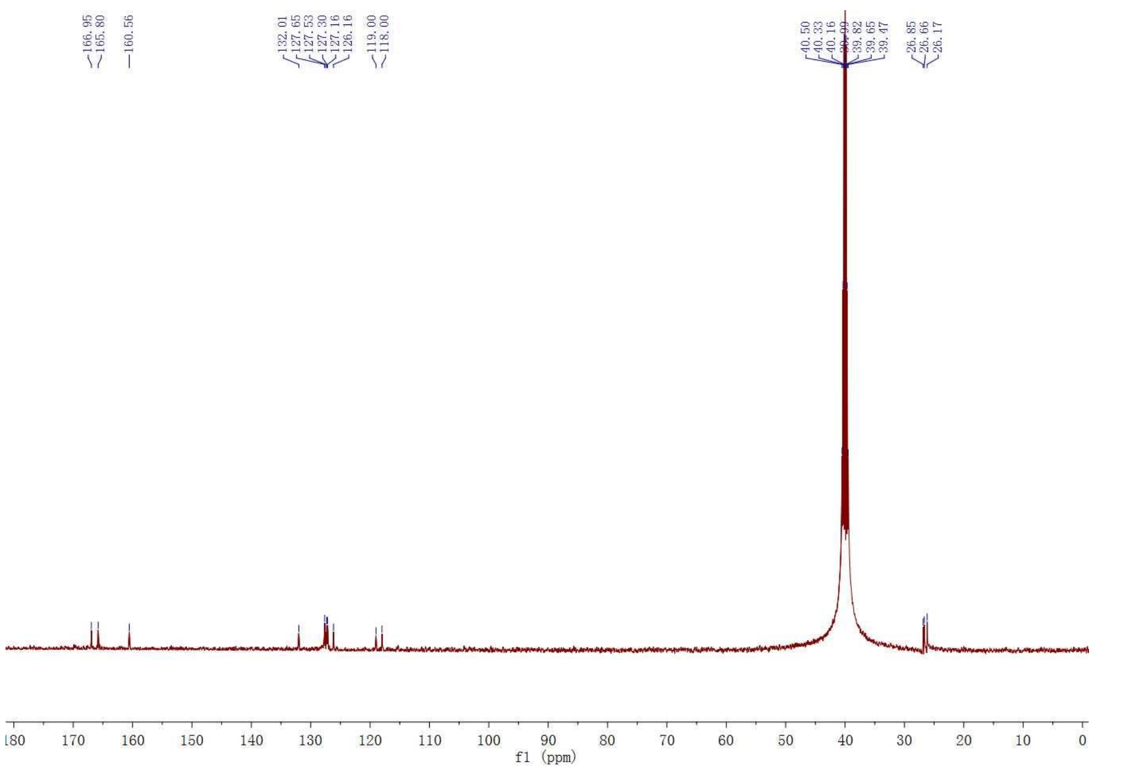

5-(4-Chlorophenyl)-N^2,N^3-diethyl-N^4-methyl-1H-pyrrole-2,3,4-tricarboxamide (**13k**)

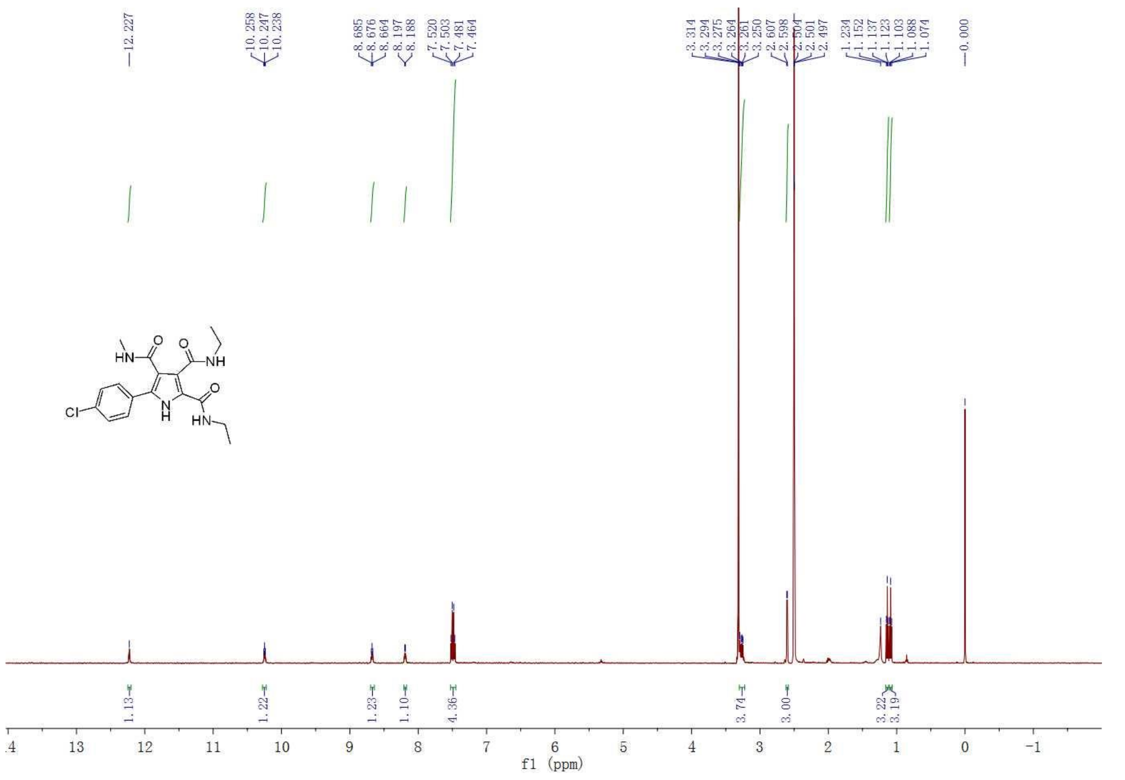

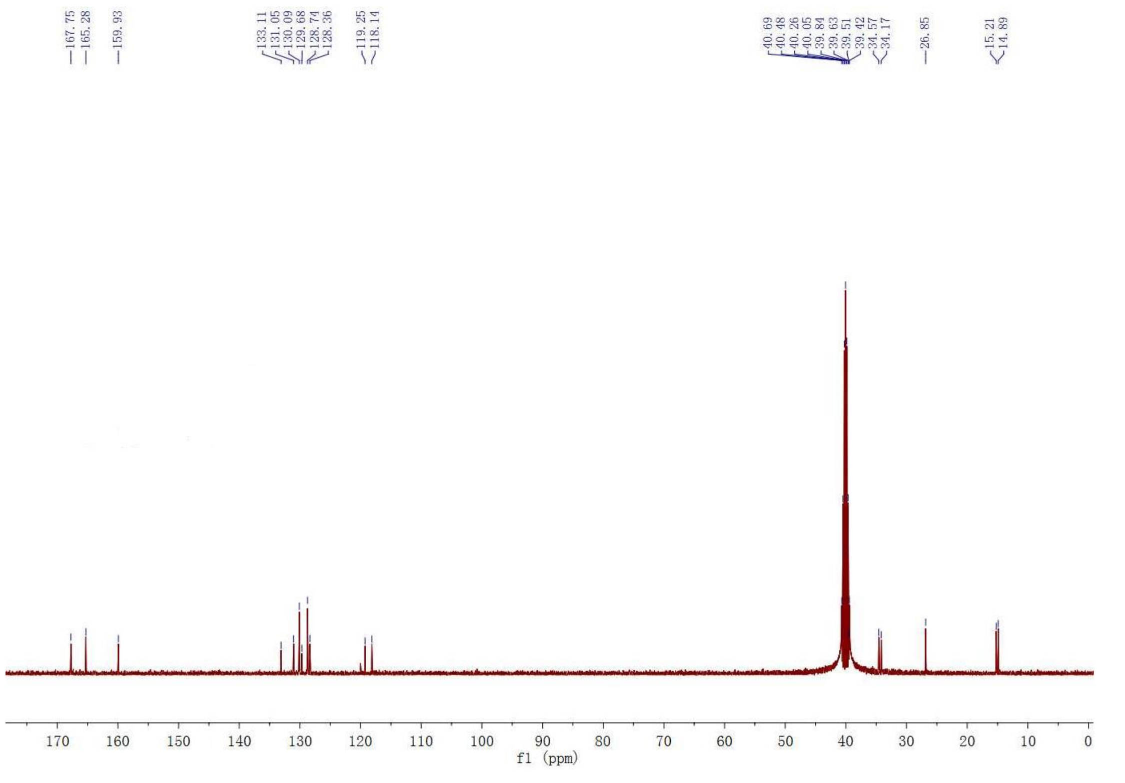

N^4-Ethyl-N^2,N^3-dimethyl-5-phenyl-1H-pyrrole-2,3,4-tricarboxamide (**13l**).

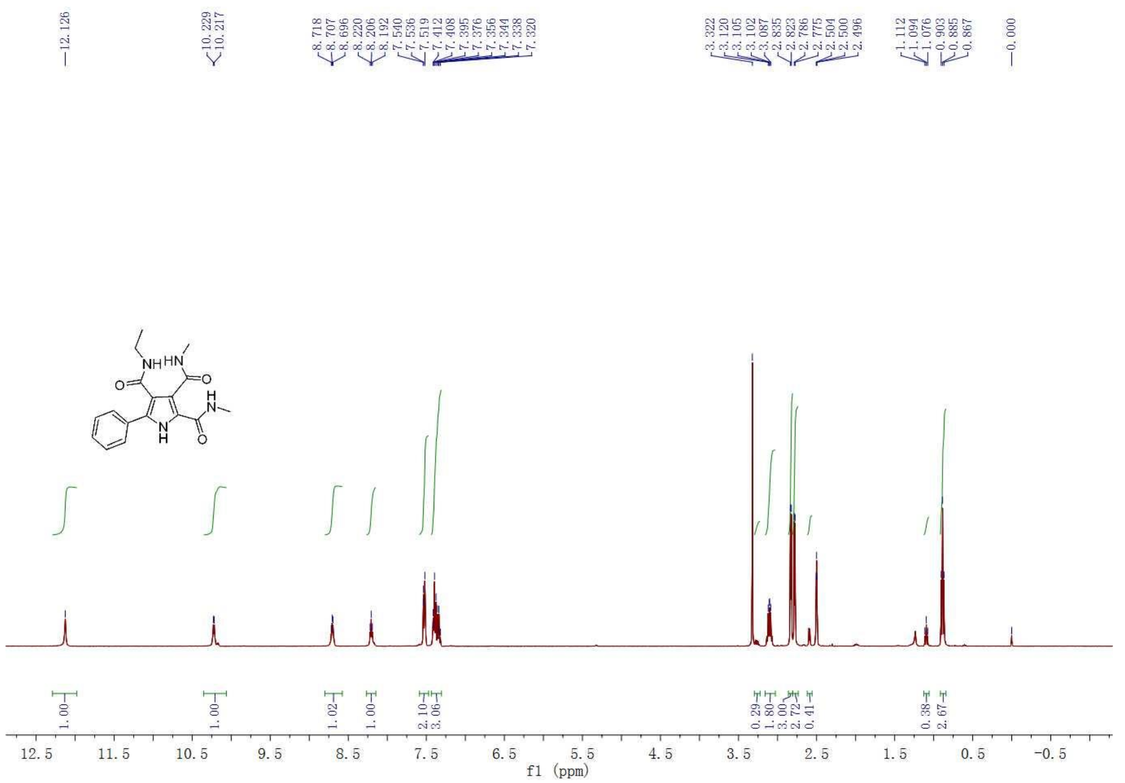

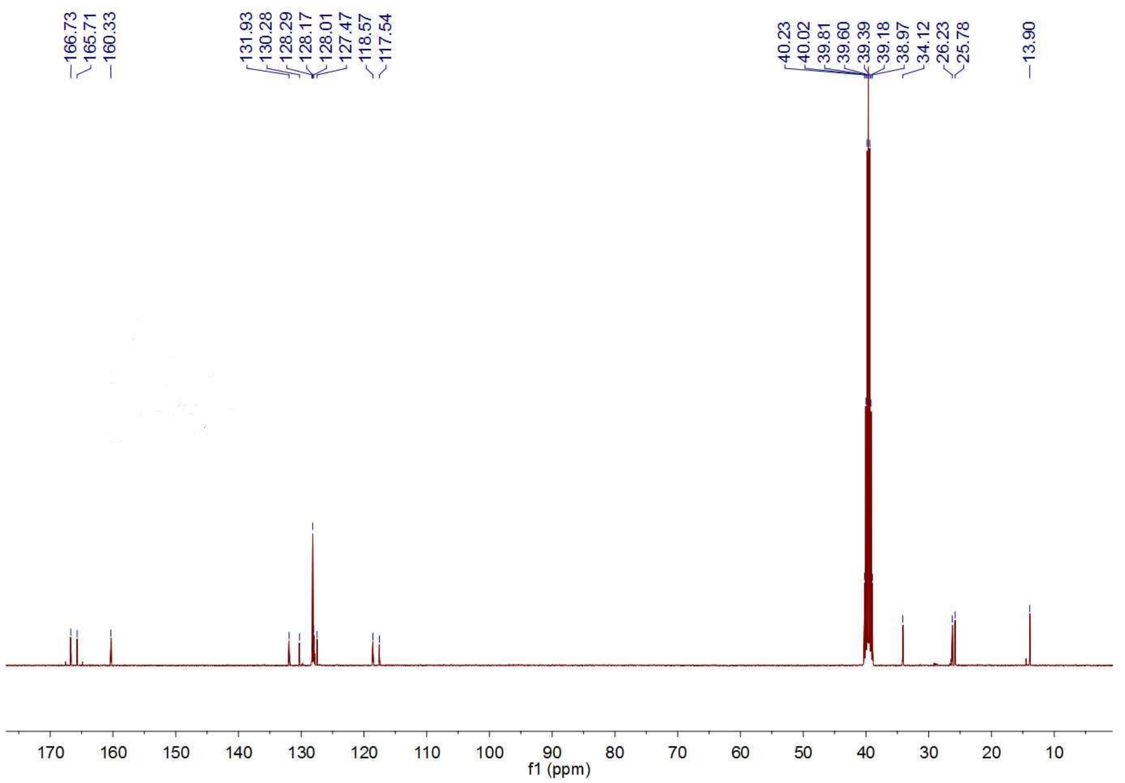

Dimethyl 4-(methylcarbamoyl)-5-phenyl-1*H*-pyrrole-2,3-dicarboxylate (**13m**)

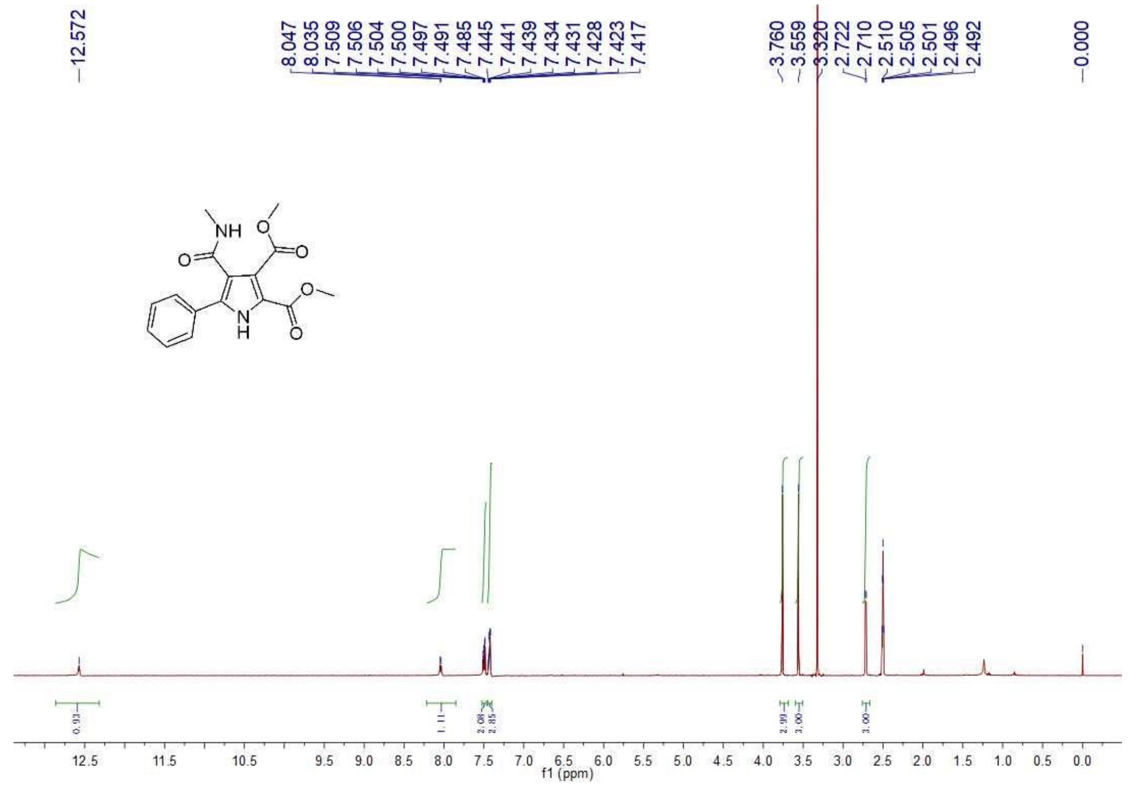

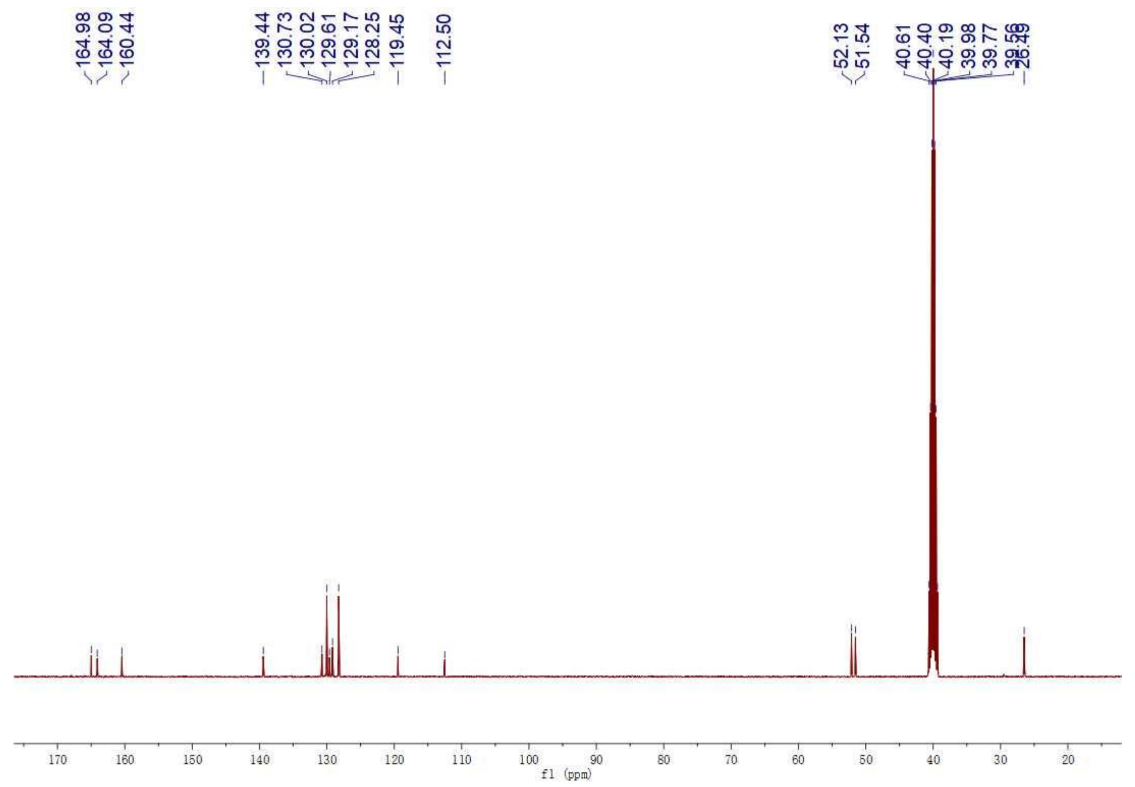

Diethyl 4-(methylcarbamoyl)-5-phenyl-1*H*-pyrrole-2,3-dicarboxylate (**13n**)

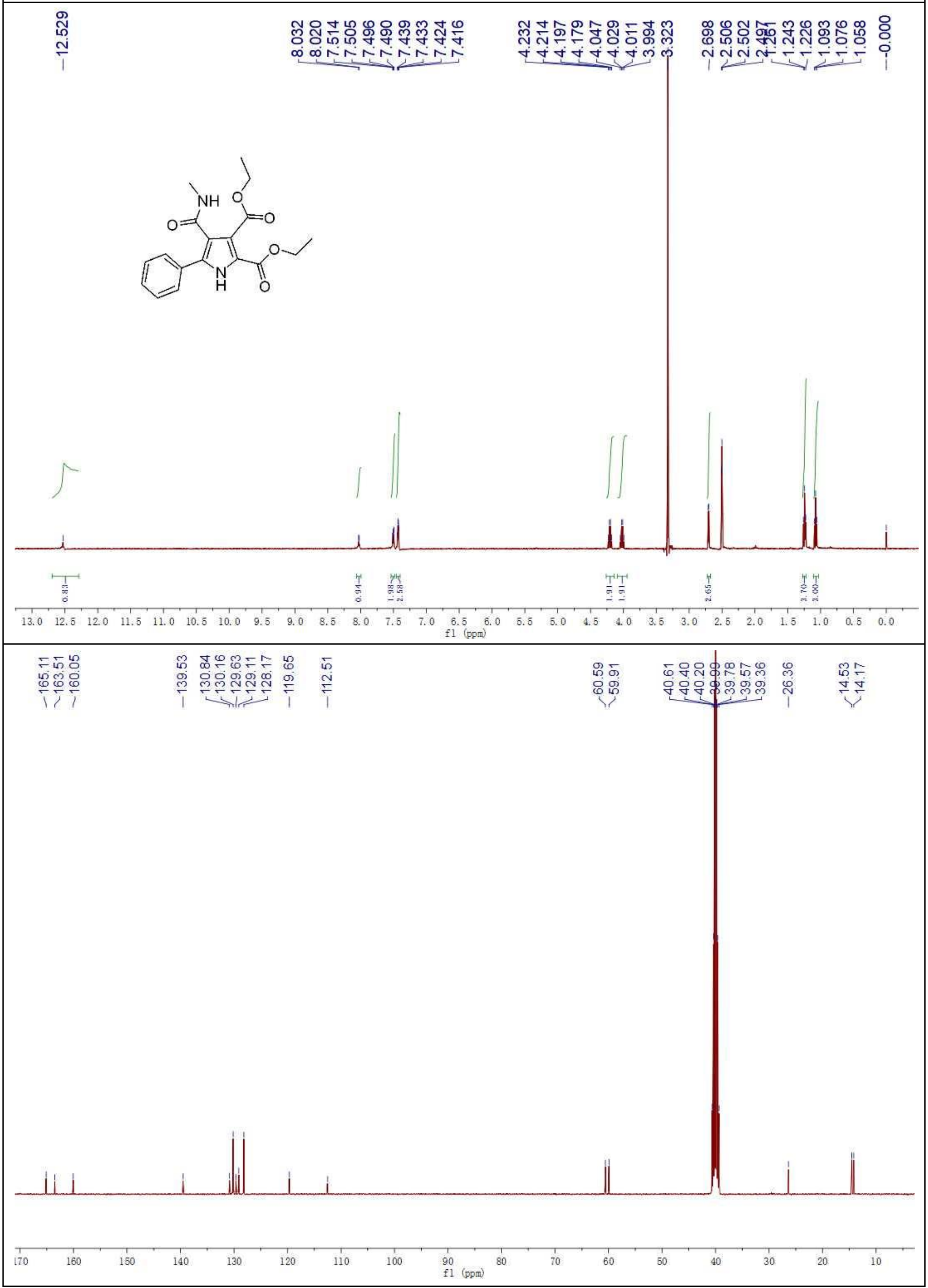

NOESY spectroscopy of compound 13k

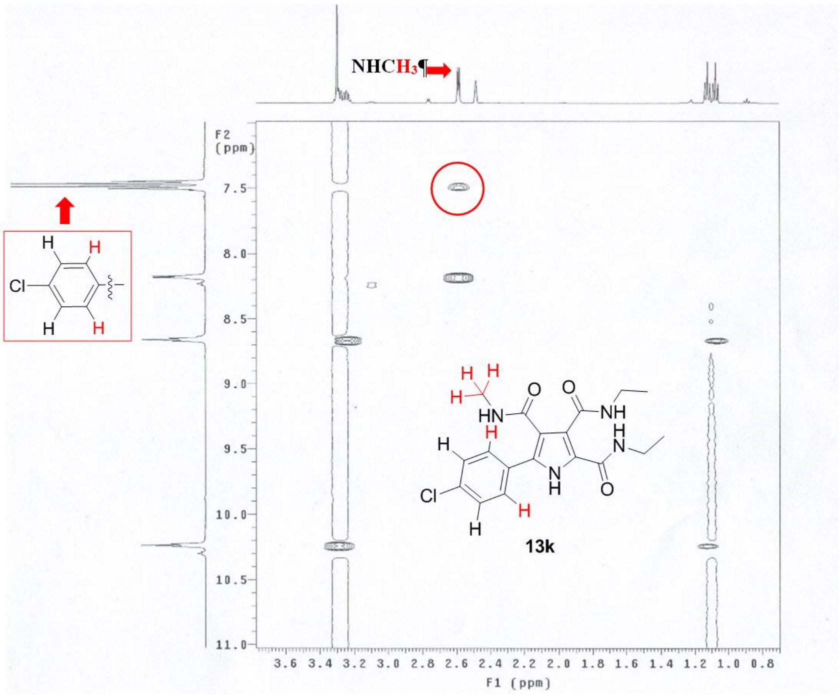

Supplement: File 1 — General information, experimental details, characterization data, copies of 1H and 13C NMR spectra of 12a–k and 13a–n, and NOESY spectra of compound 13k. [file Beilstein_J_Org_Chem-14-884-s001.pdf]
